# Sirtuin Upregulation in the Cochlea During Honokiol-Mediated Protection Against Cisplatin Ototoxicity

**DOI:** 10.3390/ph19101618

**Published:** 2026-10-12

**Authors:** Devin Thomas, Yingjie Zhou, Chail Koo, Lexie Kessler, Prerita Pandya, Robert J. Fuentes, Alan Robinson, Shreya Sriram, Pranit Guntupalli, Claus-Peter Richter, Xiaodong Tan

**Affiliations:** 1 Department of Otolaryngology-Head and Neck Surgery, Feinberg School of Medicine, Northwestern University, Chicago, IL 60611, USA; devin.thomas@northwestern.edu (D.T.); chail.koo@northwestern.edu (C.K.); lexie.kessler@northwestern.edu (L.K.); prerita.pandya@northwestern.edu (P.P.); robert.fuentes@northwestern.edu (R.J.F.); alan.robinson@northwestern.edu (A.R.); ssriram7@jhmi.edu (S.S.); pranitguntupalli@gmail.com (P.G.); 2 Department of Communication Sciences and Disorders, Northwestern University, Evanston, IL 60208, USA; yingjie.zhou@northwestern.edu; 3 Knowles Hearing Center, Northwestern University, Evanston, IL 60208, USA; 4 Department of Biomedical Engineering, Northwestern University, Evanston, IL 60208, USA

**Keywords:** cisplatin-induced hearing loss, tumor-bearing mice, chemotherapy, auditory brainstem response, RT-qPCR, cancer treatment

## Abstract

**Background/Objectives:** Cisplatin-induced hearing loss (CIHL) is a major global health sequela of chemotherapy, affecting over half a million cancer patients annually. We previously reported that honokiol, a multifunctional molecule compatible with cisplatin, protects against CIHL and upregulates sirtuin 3. The sirtuin family is composed of seven highly conserved yet differentially expressed members that are critical enzymes for oxidative stress regulation in different cellular compartments. Therefore, this study aims to further verify the expression and upregulation of the entire sirtuin family during honokiol-mediated hearing protection against CIHL in a tumor-bearing mouse model. **Methods:** Adult female mice carrying the mouse mammary tumor virus polyomavirus middle T agent oncogene were used. These mice developed palpable tumors spontaneously at the age of 70.8 ± 10.3 days. CIHL was induced using a three-cycle (14 days/cycle) low-dose (4 mg/kg/day for 4 days per cycle, intraperitoneal injection) cisplatin treatment mimicking chemotherapy regimens. Honokiol (5–20 mg/kg) was administered one hour before cisplatin. Cochlear function was evaluated by auditory brainstem response threshold shifts and synaptic changes. Immunostaining and RT-qPCR were performed to verify the upregulation of sirtuin family members (1, 2, 3, 5, and 7) during these processes. **Results:** CIHL was observed in the high-frequency range (24–32 kHz), which was mitigated by honokiol treatment. The optimal dose of honokiol for oto-protection and animal survival was 10 mg/kg. Fluorescence signals of sirtuins 1, 2, 3, 5, and 7 increased in cochleae from honokiol-treated mice. RT-qPCR confirmed a significant upregulation of sirtuin 3 mRNA with honokiol treatment. **Conclusions:** Sirtuin family upregulation was involved in honokiol-mediated hearing protection against CIHL. The dose–response curve of honokiol lays the foundation for potential translational applications for human studies.

## 1. Introduction

Cisplatin is a potent, widely used platinum-based chemotherapeutic agent [1]. However, it is also a well-recognized ototoxic compound, with an incidence of hearing loss close to or over 50% in treated patients, varying with age, dose, and treatment regimen [2,3,4]. In recent review studies, cisplatin-induced hearing loss (CIHL) is identified as the predominant cause of hearing loss during chemotherapy [5], leading to an estimated half a million new cases globally each year [6]. CIHL causes cellular and structural damage throughout the cochlea, with outer hair cells (OHCs) being particularly vulnerable [7,8]. The underlying mechanisms are primarily attributed to excessive generation of reactive oxygen species (ROS) [9,10,11], which causes the depletion of intrinsic antioxidants (e.g., glutathione) and activation of multiple cell death pathways [12,13]. Conventional protective strategies, such as the use of exogenous antioxidants as free radical scavengers [14,15,16], raise concerns of deactivating cisplatin [17,18,19] and protecting tumor cells [20]. Currently, sodium thiosulfate is the only approved clinical intervention for CIHL, but its use is limited to pediatric patients, with the known risk of reducing cisplatin’s antitumor effect [21].

Sirtuins represent a highly conserved family of NAD^+^-dependent deacetylases critical for metabolic regulation and oxidative stress defense [22,23,24]. In mammals, seven sirtuin family members (Sirt1–7) are differentially expressed in the cytoplasm (Sirt1 and Sirt2), mitochondria (Sirt3–5), and nucleus (Sirt1, 2, 6, 7) [23,25,26,27], forming a network for precise control of diverse physiological processes. Through modulating ROS, sirtuins play critical roles in various biological processes, including metabolism [28], longevity [25,29], and carcinogenesis [29]. These versatile activities highlight the broader therapeutic potential of sirtuins as targets for intervention in various disease models. For example, pharmacological upregulation of Sirt1 attenuates cisplatin-induced pulmonary [30] and vascular [31] injury through the activation of nuclear factor erythroid 2-related factor 2 (Nrf2) pathway. Similarly, activation of Sirt3 is critical for maintaining mitochondrial redox homeostasis in cisplatin-induced hair cell loss in zebrafish [32] and cardiotoxicity in rats [33]. In addition, sirtuins are also therapeutic targets for cancer [34] and other diseases, as summarized in a recent review [35].

Sirtuins are also expressed in cochlear cells [36]. However, the roles of sirtuins in hearing protection remain poorly understood despite their critical regulatory functions [37]. We previously showed that Sirt3 was upregulated during hearing protection against cisplatin ototoxicity using honokiol [38], a naturally occurring biphenolic lignan found in the root and bark of the genus *Magnolia* [39]. Honokiol is a multifunctional molecule and a reported Sirt3 activator exhibiting a broad range of pharmacological activities, including anti-inflammatory, antioxidant, antitumor [40,41], and normal tissue protective effects [42,43,44]. It is an approved dietary supplement in Europe and was recently tested in combination with chemotherapeutic drugs to enhance anticancer efficacy [45,46,47]. These studies suggest that honokiol is a promising candidate for treating CIHL, and Sirt3 might be involved in the mechanism of this honokiol-mediated protection. We have also shown that Sirt5 is upregulated by honokiol in an early-onset hearing loss mouse model due to Sirt3 knockout [48]. Given the high conservation in sequence and function among the Sirtuin family members, it is logical to hypothesize that upregulation by honokiol is not limited to Sirt3 and Sirt5 but rather involves the broader sirtuin network in different cellular compartments. In this study, we thoroughly investigated this honokiol-mediated hearing protection against CIHL in a tumor-bearing mouse model, further examined the expression of multiple sirtuin family members in this process, and explored potential functional pathways involving sirtuins during this process.

## 2. Results

### 2.1. Honokiol Ameliorates CIHL in a Tumor-Bearing Mouse Model

#### 2.1.1. Significant CIHL Was Induced by a Three-Cycle Cisplatin Treatment Regimen

**Figure 1:**
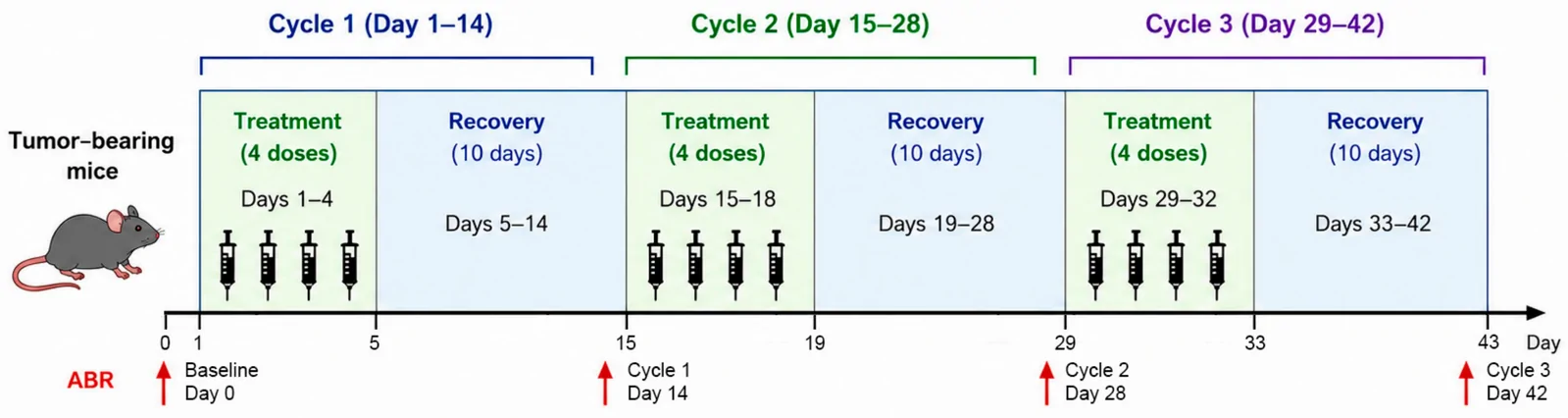
Schematic image showing the three-cycle low-dose chemotherapy regimen. Treatment commenced when the total tumor volume reached 500 mm^3^. The dose of cisplatin was 4 mg/kg/day, or 16 mg/kg/cycle, with a total amount of 48 mg/kg after three cycles. Honokiol was administered one hour prior to cisplatin, with three different daily doses (5, 10, and 20 mg/kg). Both cisplatin and honokiol were delivered through i.p. injections, and vehicles (saline or corn oil) were given as controls whenever applicable.

**Figure 2:**
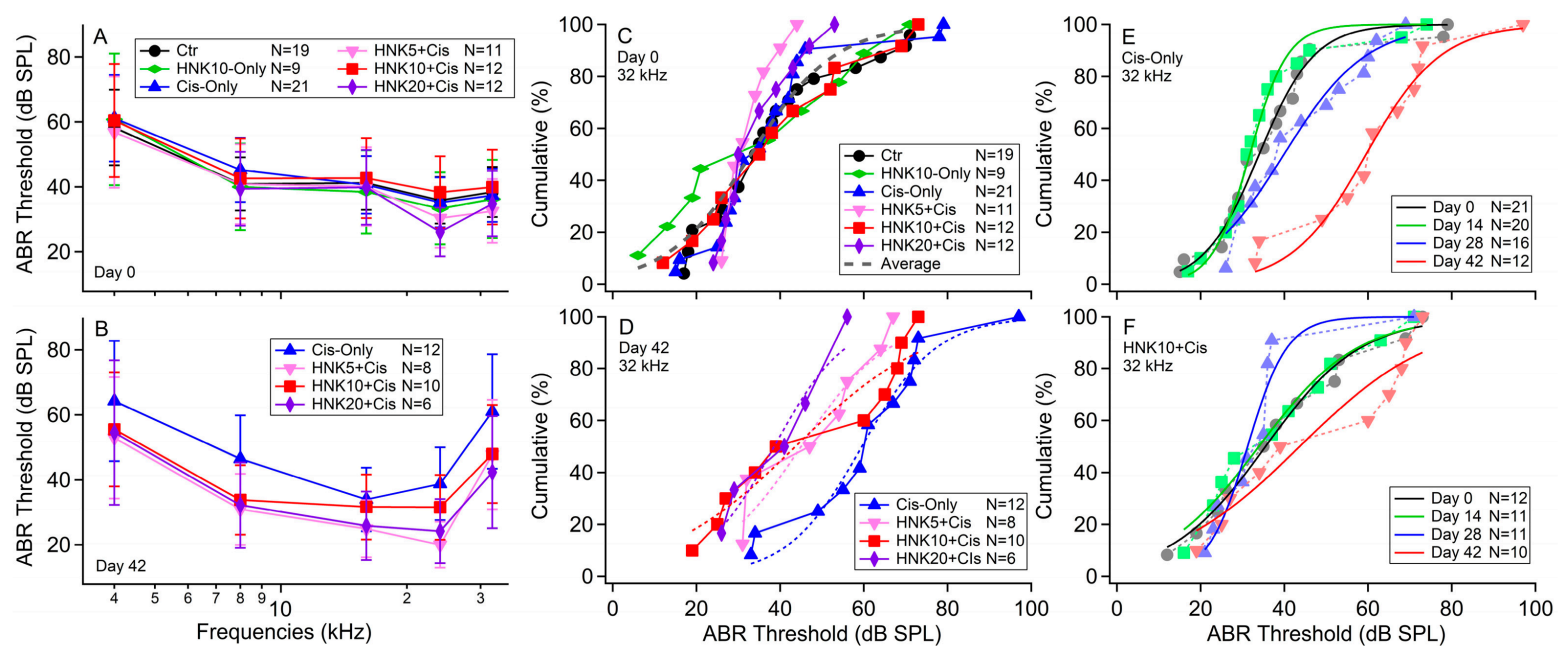
ABR threshold measured before, during, and after the three-cycle chemotherapy regimen. (**A**) Baseline (Day 0) of all the groups measured before the treatment. (**B**) End of the study (Day 42) of all surviving animals. Note that the rest of the animals were removed from the study due to oversized tumor growth or health issues. (**C**) Cumulative percentage plots of the data from all the groups at 32 kHz on Day 0. A logistic curve fitting (dashed curve) was performed, from which an xhalf (average threshold) of 37.4 ± 2.4 dB SPL was obtained. (**D**) Cumulative percentage plots and their curve fittings of the surviving animals in the four treatment groups at 32 kHz on Day 42. (**E**) Progression of the threshold shift over each cycle of the Cis-only group at 32 kHz. (**F**) Progression of the threshold shifts over each cycle of the HNK10+Cis group at 32 kHz. Ctr: Control; HNK: honokiol; Cis: cisplatin. The doses of honokiol (5, 10, 20 mg/kg) were indicated by the names of the treatment groups. Data presented in (**A**,**B**): mean ± standard error.

Transgenic mice carrying mouse mammary tumor virus polyomavirus middle T agent oncogene (MMTV-PyMT, Jax Stock #: 002374, FVB background) [49] were used as the cancer mouse model for this study. Female MMTV-PyMT carriers developed palpable mammary tumors reaching a volume of 500 mm^3^ at 70.8 ± 10.3 days of life (*n* = 84), which was set as the starting point for therapeutic interventions. To mimic clinical chemotherapy paradigms, mice received a three-cycle (14 days/cycle) low-dose cisplatin regimen consisting of 4 mg/kg/day for four consecutive days per cycle (Figure 1) [38,50,51], delivered through intraperitoneal (i.p.) injection. Hearing was assessed by auditory brainstem response (ABR) thresholds at frequencies from 4 to 32 kHz (Figure 2). Baseline (Day 0) ABR thresholds showed no significant difference among experimental groups (Figure 2A). After three cycles of cisplatin treatment, mice exhibited clear ABR threshold elevations across all frequencies (Figure 2B, Cis-only, the blue curve). Threshold shifts reached statistical significance at 32 kHz (*p* < 0.05), compared to the baseline of the same group. This is consistent with our previous study [38] and is comparable with the results from other studies showing that CIHL primarily affects the high-frequency range [51,52].

#### 2.1.2. CIHL Was Ameliorated by Parallel Honokiol Treatment

To verify the protective effect of honokiol against CIHL, three different doses (5, 10, and 20 mg/kg) were adopted and were given through i.p. injection one hour before each cisplatin dose. As shown in Figure 2B, CIHL was largely ameliorated by honokiol application in all three dosage groups (marked as HNK5+Cis, HNK10+Cis, and HNK20+Cis, respectively). No statistically significant ABR threshold shift was observed at any frequency in the honokiol-treated groups.

It should be noted that not all animals have completed the full treatment course. Animals with tumor size ≥ 1500 mm^3^, weight loss ≥ 25%, or severe health deterioration were removed from the study. Figure 2B includes all the mice that survived until the end of the three treatment cycles.

#### 2.1.3. Cumulative Percentage Plots Delineated Threshold Shifts in Different Groups

ABR thresholds were also plotted as a cumulative percentage to better capture data variability and to address missing data points in case of animal loss or no response to acoustic stimuli [53] (Figure 2C–F). As an example, baseline ABR thresholds at 32 kHz of all treatment groups were shown in Figure 2C. A similar distribution of the ABR thresholds was indicated by the curves, which were fitted to a logistic function. The xhalf, or the average ABR threshold depicted by the curve fitting, was 37.4 ± 16.4 dB SPL (Figure 2C, the dashed curve). After three treatment cycles (Day 42, Figure 2D), the plot shifted to the right for the animals treated with cisplatin alone (Cis-only group, the blue curve), with an xhalf of 61.0 ± 17.5 dB SPL. Expressed as threshold elevations at 32 kHz: The CIHL after the third treatment cycle (Day 42) was 23.6 ± 1.4 dB, whereas no significant elevation occurred after the first (Day 14, −1.8 ± 2.2 dB) or second (Day 28, 5.5 ± 2 dB) treatment cycles (Figure 2E). Combined cisplatin and honokiol treatment attenuated this threshold shift, with xhalfs of 47.7 ± 14.8, 47.9 ± 21, and 42.3 ± 12.9, for honokiol at 5, 10, and 20 mg/kg, respectively (Figure 2D, HNK5+Cis, HNK10+Cis, and HNK20+Cis groups). Compared to the significant cisplatin-induced threshold shifts (Figure 2E), a minor and non-significant threshold shift occurred in Cycle 3 (8.0 ± 2.0 dB, *p* > 0.05) in the HNK10+Cis group (Figure 2F).

ABR threshold data measured in all three cycles during the chemotherapy regimen were plotted in Figure S1. Cumulative percentage plots of ABR threshold changes at 24 kHz can be found in Figure S2, and the rest in the folder for the raw data shared via the link provided at the end of the paper. A significant threshold change was also observed at 24 kHz on Cycle 3 (Day 42) between Cis-only and HNK5+Cis (*p* < 0.05) and between Cis-only and HNK20+Cis (*p* < 0.05) groups. However, no significant changes were detected within the Cis-only group across different cycles at 24 kHz. In addition, no significant threshold shift was observed at any other frequencies in any group. Overall, the results showed that honokiol treatment significantly mitigated CIHL, which was presented at later stages of treatment at high frequencies.

Since the mice in the control and honokiol alone-treated groups did not survive till Cycle 3 due to uncontrollable tumor growth, proper control groups were missing in these MMTV-PyMT mice. Therefore, a group of female FVB wild-type mice treated with honokiol at a dose of 20 mg/kg/day (HNK20-Only, *n* = 8) or a vehicle (*n* = 6) was used as the healthy control, which was also included in Figure S1. Honokiol treatment alone did not affect ABR threshold during the three-cycle regimen.

### 2.2. Honokiol Mitigates Cisplatin-Induced Systemic Toxicity While Preserving Anti-Tumor Efficacy

#### 2.2.1. Honokiol Improves Survival Rate and Prevents Weight Loss in Animals Undergoing Cisplatin Chemotherapy

**Figure 3:**
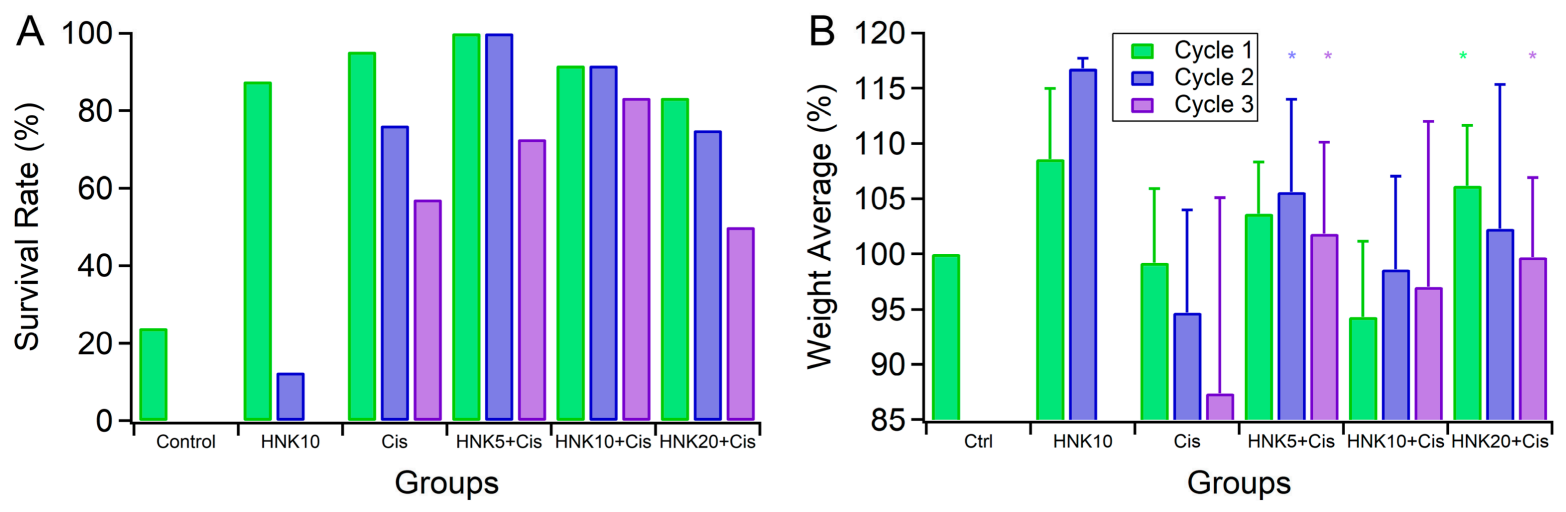
Impact of cisplatin and honokiol co-treatment on animal survival and weight change. (**A**) Animal survival rates recorded at the end of each treatment cycle. (**B**) Average animal weight changes in each cycle, normalized to baseline values measured immediately prior to treatment (Day 0). Data presented in (**B**): mean ± standard deviation (SD). * *p* < 0.05, compared to the Cis-only group.

Cisplatin treatment could induce severe weight loss, which can be up to 40% in some studies [54]. In our study, animals that lost more than 25% of their weight or that reached moribund conditions were removed from the study and euthanized for humane reasons. This systemic toxicity, combined with tumor oversize criteria, resulted in a low overall survival rate, with only 57.1% (12 of 21) of the animals completing the third treatment cycle in the Cis-only group (Figure 3A). Among the nine prematurely terminated cases, the majority (six) were due to weight loss or moribund, while three were due to oversized tumors. Furthermore, the surviving animals in the Cis-only group displayed a cycle-by-cycle physical deterioration, with the average body weight dropping to 81.0 ± 14.9% of their pre-treatment baseline by the end of the third cycle (Figure 3B).

Co-treatment with honokiol largely prevented this cisplatin-induced physical decline. By the end of the third cycle, survival rates for the honokiol co-treated groups were 72.7% (8 of 11), 83.3% (10 of 12), and 50% (6 of 12) for honokiol 5, 10, and 20 mg/kg, respectively (Figure 3A). The survival rates of all the groups by the end of each cycle were summarized in Table S1. The data indicated that the 10 mg/kg dosage of honokiol provided the optimal protection for animal survival. The loss of body mass was also alleviated in the surviving animals, with average weights at the conclusion of the study maintained at 101.8 ± 8.3%, 97.0 ± 15.0%, and 101.0 ± 5.7%. Since the group sizes were relatively small and unequal, a more conservative Welch’s ANOVA was performed (F(3,12.43) = 4.05, *p* = 0.0323). A following Games–Howell post hoc test showed that the weight losses were significantly lower in honokiol co-treated groups. Significant differences were detected in Cycle 1 (*p* = 0.025 for HNK20+Cis group), Cycle 2 (*p* = 0.026 for HNK5+Cis group), and Cycle 3 (*p* = 0.024 and 0.028 for HNK5+Cis and HNK20+Cis groups, respectively) when compared to the Cis-only group (Figure 3B). The values of animal weight during the treatment were reported in Figure S3. No significant difference in pre-treatment baseline weight was detected in different treatment groups.

The Kaplan–Meier survival analysis [55] was not performed because the exact timing of individual animal losses was not recorded consistently throughout the study. Instead, survival of the four cisplatin-treated groups at the end of Cycle 3 was compared using a four-group contingency analysis. Although the proportion of surviving animals was numerically higher in the honokiol co-treatment groups than in the Cis-only group, the overall difference among groups was not statistically significant (χ^2^(3) = 1.36, *p* = 0.715).

#### 2.2.2. Honokiol Is Compatible with Cisplatin in Tumor Suppression

Interference with the therapeutic effects of cisplatin is one of the biggest concerns for CIHL treatment during chemotherapy. We have reported in a previous publication that co-treatment of honokiol and cisplatin exerts further tumor suppression based on limited data [38]. Adding to the results, further studies on tumor size measurement were also performed on a subset of the animals as shown in Figure S4. In the control group, tumors grew rapidly from 500 mm^3^ to the 1500 mm^3^ euthanasia threshold within 6.5 ± 1.2 days (*n* = 4, the black traces). Honokiol alone delayed this progression by doubling the time required (13.3 ± 3.2 days, *n* = 4, the green traces) to reach the maximum tumor burden, indicating that honokiol alone has an antitumor effect.

As expected, cisplatin alone had a prevailing effect on tumor growth (*n* = 6, the blue traces). Although cisplatin did not fully cure the tumor, it reduced the tumor size during the treatment period of each cycle. However, tumor growth restarted during the recovery period, and tumor size increased significantly by the end of Cycle 2 (Day 28) and Cycle 3 (Day 42) to about 150% of the original size.

Co-treatment of honokiol (10 mg/kg) and cisplatin further suppressed tumor growth (*n* = 7, the red traces), as the total tumor size was always smaller than that of the Cis-only group on each given date of the study. At the end of each cycle, the tumor size was 50%, 110%, and 110%, respectively. Further tumor suppression was also shown in animals co-treated with honokiol at dosages of 5 (*n* = 2) and 20 mg/kg (*n* = 4), although the full record of tumor growth was lost with these animals. These results indicated an additional antitumor effect introduced by honokiol, since the co-treatment exerts further tumor suppression power. This enhancement from honokiol has also been proven frequently in previous studies with different in vitro and in vivo models and different cancer types [56,57].

### 2.3. Honokiol Protects IHC Ribbon Synapses During Cisplatin Treatment

#### 2.3.1. Cisplatin Induces the Degeneration of Ribbon Synapses

**Figure 4:**
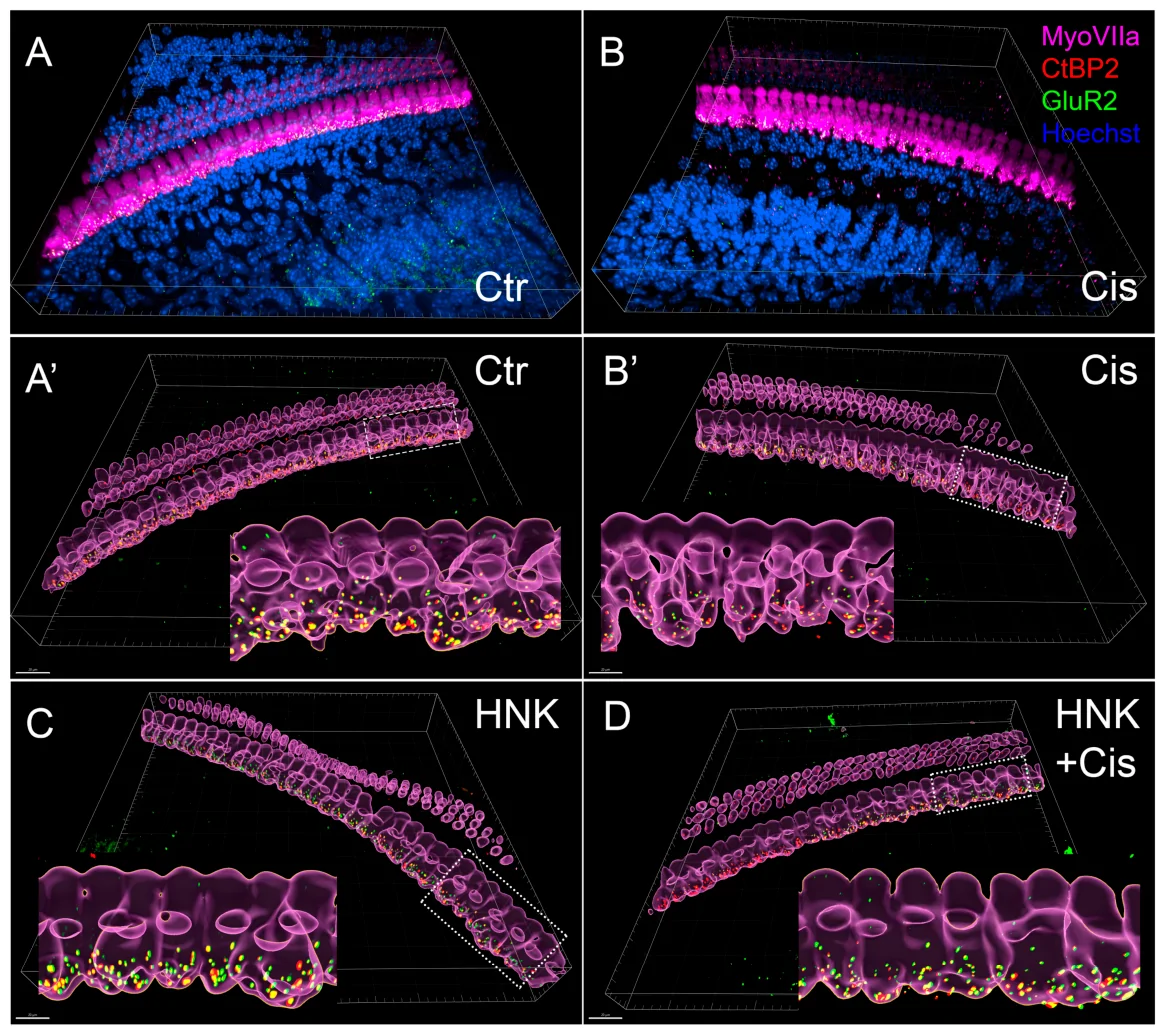
Confocal images showing the changes in ribbon synapses after different treatments. (**A**) and (**B**) Representative confocal image stacks of the Ctr and Cis-only groups. (**A′**–**D**) Processed images showing only the hair cells and synaptic puncta at the bottom region of the IHCs. All images were taken from cochlear samples at the frequency range of 9.5–19.1 kHz. Ctr: control; Cis: cisplatin 4 mg/kg; HNK: honokiol 10 mg/kg. The insets are enlarged from the rectangular area in each panel (estimated frequency range: 15–19 kHz). Scale bars: 20 µm.

OHC loss is the most frequently observed change in CIHL, which is well-documented and has also been reported in our previous study. In the current study, we further investigated the impact of the three-cycle cisplatin regimen on IHC ribbon synapses. Synaptic integrity was evaluated using immunostaining combined with 3D confocal microscopy on harvested cochlear samples. Figure 4 shows the representative confocal images of cochlear samples from different groups following three cycles of treatment. Puncta of C-terminal binding protein 2 (CtBP2, pre-synaptic component, shown in red) and glutamate receptor 2 (GluR2, post-synaptic component, green) were identifiable at the bottom of the IHCs. Most CtBP2 and GluR2 puncta were colocalized in the untreated mice (Figure 4A). Cisplatin treatment caused a decrease in both the number and size of the puncta (Figure 4B). For quantitative studies, IHCs and their synapses were digitally separated (Figure 4A′–D). All samples in Figure 4 were harvested from the animals treated in parallel, from the same cochlear segment (frequency range: 9.5–19.1 kHz [38]) and processed in parallel during immunostaining and confocal imaging. An enlarged area at the basal end (around 15–19 kHz) of each sample was shown in the inset of each panel.

**Figure 5:**
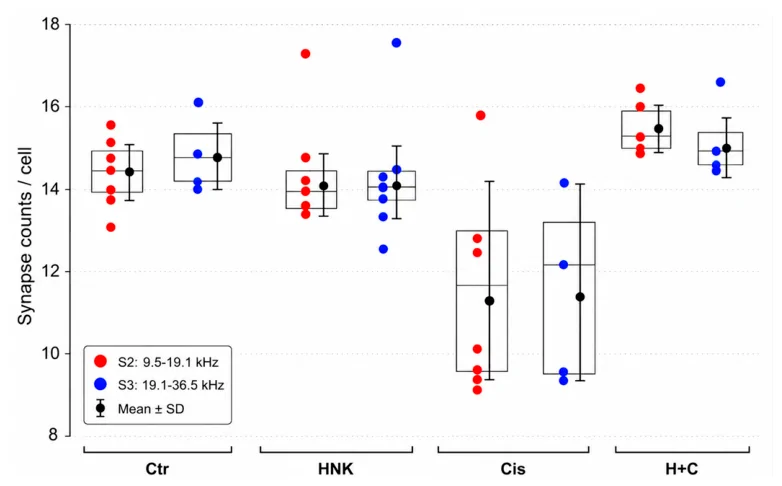
Box–and–whisker plots showing the distribution of ribbon synapse counts per IHC in control (Ctr), honokiol-treated (HNK), cisplatin-treated (Cis), and honokiol plus cisplatin-treated (H+C) groups after three cycles of treatments. Synapse counts were quantified in two cochlear frequency regions: S2 (9.5–19.1 kHz; red symbols) and S3 (19.1–36.5 kHz; blue symbols). Each dot represents the average counts from one sample. Box plots indicate the median (center line), interquartile range (box), and minimum and maximum values (whiskers). Mean ± SDs were also plotted. Cisplatin treatment significantly reduced synapse counts compared with control animals, whereas honokiol co-treatment preserved synapse counts and prevented cisplatin-induced synaptopathy. Statistical comparisons were performed between the pooled control and cisplatin groups and between the pooled cisplatin and honokiol plus cisplatin groups.

Synapse counting and statistics were performed on two cochlear regions, segment 2 and 3 (S2 and S3), which correspond to frequency ranges 9.5–19.1 and 19.1–36.5 kHz, respectively (Figure 5) [38]. CtBP2 and GluR2 colocalized puncta at the bottom region of IHCs were considered intact synapses (Figure 4, the insets showing the enlarged IHCs), and each dot in Figure 5 represents the average number of intact synapses per IHC from one sample. Mice in the control group contained an average of 14.94 ± 0.78 and 15.58 ± 0.54 per IHC in the S2 and S3 regions, respectively (Figure 4A′ and Figure 5, Ctr). After three cycles of cisplatin treatment, a significant decrease in synaptic counting was observed (11.90 ± 2.10 and 12.19 ± 2.18, respectively; *p* = 0.00023, compared with controls) (Figure 4B′ and Figure 5, Cis).

#### 2.3.2. Honokiol Prevents the Loss of Ribbon Synapses Induced by Cisplatin

Honokiol treatment alone did not alter the number of synapses (S2: 15.06 ± 1.28; S3: 14.80 ± 1.20; *p* = 0.73), indicating that honokiol itself had no detectable effect on normal cochlear synaptic integrity (Figure 4C and Figure 5, HNK). However, co-administration of honokiol substantially preserved ribbon synapses following cisplatin treatment. Synapse counts increased to 15.49 ± 0.50 and 15.25 ± 0.75, respectively, in S2 and S3 (*p* = 9.4 × 10^−5^, compared with the Cis-only group) (Figure 4D and Figure 5, H+C). The numbers were comparable to untreated controls or the honokiol-only treated group (*p* = 0.31). These results suggested that honokiol largely prevented cisplatin-induced cochlear synaptopathy.

#### 2.3.3. Cisplatin Affects Both Pre- and Post-Synaptic Components, Which Were Both Rescued by Honokiol

To determine whether pre- and post-synaptic components could be affected by cisplatin and honokiol treatments differently, we counted the puncta for CtBP2 and GluR2 separately in a portion of the samples. As plotted in Figure S5, both pre- (CtBP2, shown in red) and post-synaptic components (GluR2, shown in green) of the puncta decreased by around 20% due to cisplatin treatment, with the changes in CtBP2 significant (*p* = 0.029) but not GluR2. This is potentially due to the high variation in GluR2 puncta counting. Honokiol co-treatment rescued both pre- and post-synaptic component loss.

### 2.4. Sirtuin Expression in the Cochlea Is Affected by Honokiol and Cisplatin Treatment

Our recent studies demonstrated that honokiol treatment upregulates both Sirt3 and Sirt5 in the cochlea in wild-type littermates of a Sirt3 knockout mouse model [48]. Given the high sequence conservation among sirtuin family members, we sought to investigate the broader expression profile of sirtuin family members in the cochlea and the influence of cisplatin and honokiol using immunostaining. To maximize the observation of potential therapeutic effects, cochleae were harvested on Day 5, following four doses of treatment in Cycle 1, from four treatment groups: Control, Cis-only, HNK10-only, and HNK10+Cis.

#### 2.4.1. Upregulation of Mitochondrial Sirtuins by Honokiol

**Figure 6:**
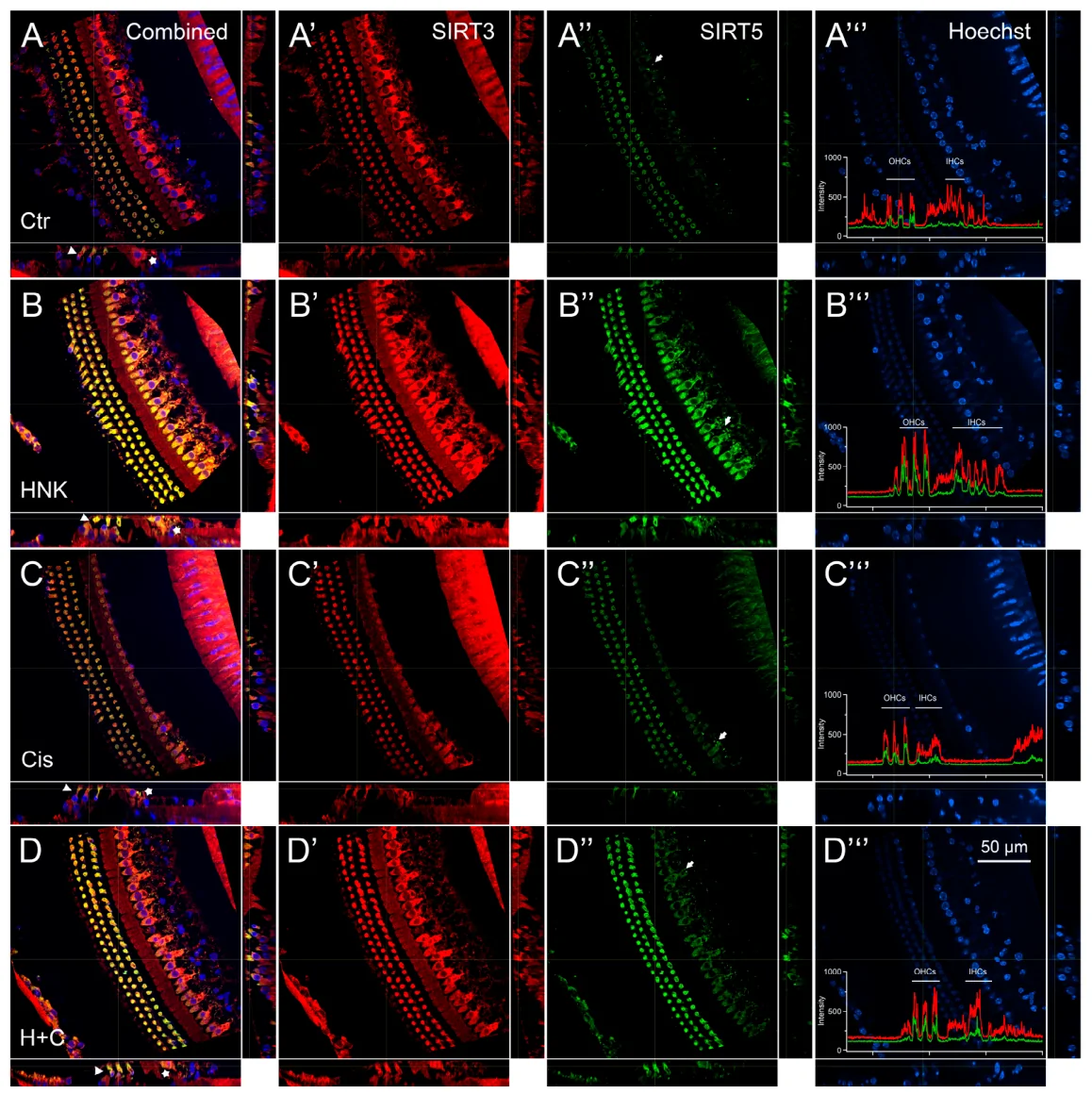
Confocal images showing Sirt3 and Sirt5 expression in the cochlea (frequency range: 19.1–36.5 kHz) after different treatments. (**A**) Control; (**B**) HNK10-only; (**C**) Cis-only; and (**D**) HNK10+Cis. For each row, the combined image and the three separate channels are presented. (**A′**–**D′**) Sirt3, (**A″**–**D″**) Sirt5, and (**A‴**–**D‴**) Hoechst. In each panel, surface (xy plane) and two orthogonal views (right: yz plane; bottom: xz plane) are presented. Surface plots are focused on the cell body of the OHCs and IHCs. Xz orthogonal views cross over three OHCs (white triangles) and one IHC (white stars). Sirt3 and Sirt5 were both expressed and upregulated by honokiol and colocalized (showing yellow) in the OHCs and IHCs in (**B**,**D**). White arrows show the bright puncta of Sirt3 and Sirt5 at the bottom regions of IHCs. Insets in the last panel of each row show the fluorescence intensity of Sirt3 (red) and Sirt5 (green) at the lines across OHC and IHC regions in the surface plot and the xz orthogonal view. All images were taken from cochlear samples at a frequency range of 19–36 kHz. Note that fluorescence intensity is the original value obtained from unprocessed confocal images, and that baseline fluorescence signal is identical in all four samples.

We first verified the expression of the two primary mitochondrial sirtuins, Sirt3 and Sirt5, in the cochlea (Figure 6). In control samples, fluorescence signals for Sirt3 (red) and Sirt5 (green) and their colocalization (yellow) were observed (Figure 6A–A‴). These fluorescence signals were markedly increased following honokiol treatment both alone (10 mg/kg) (Figure 6B–B‴) and in combination with cisplatin (Figure 6D–D‴). Cisplatin treatment alone did not affect the fluorescence signal of either Sirt3 or Sirt5 (Figure 6C–C‴). Despite their coordinated upregulation, the expression patterns of Sirt3 and Sirt5 exhibited subtle differences. While increased Sirt3 fluorescence signal was also observed in supporting cells (SCs), increased Sirt5 signal was mostly observed in hair cells, as shown in the xz orthogonal views in the panels for Sirt3 and Sirt5. Additionally, bright puncta of both Sirt3 and Sirt5 were consistently observed at the basal part of the IHCs, as indicated by the white arrows.

While Sirt3 and Sirt5 are expected to be expressed in the mitochondria, their precise subcellular localization could not be resolved by confocal microscopy, as also shown in other studies related to mitochondrial localization (e.g., [58,59]).

#### 2.4.2. Upregulation of Nuclear Sirtuin by Both Cisplatin and Honokiol

**Figure 7:**
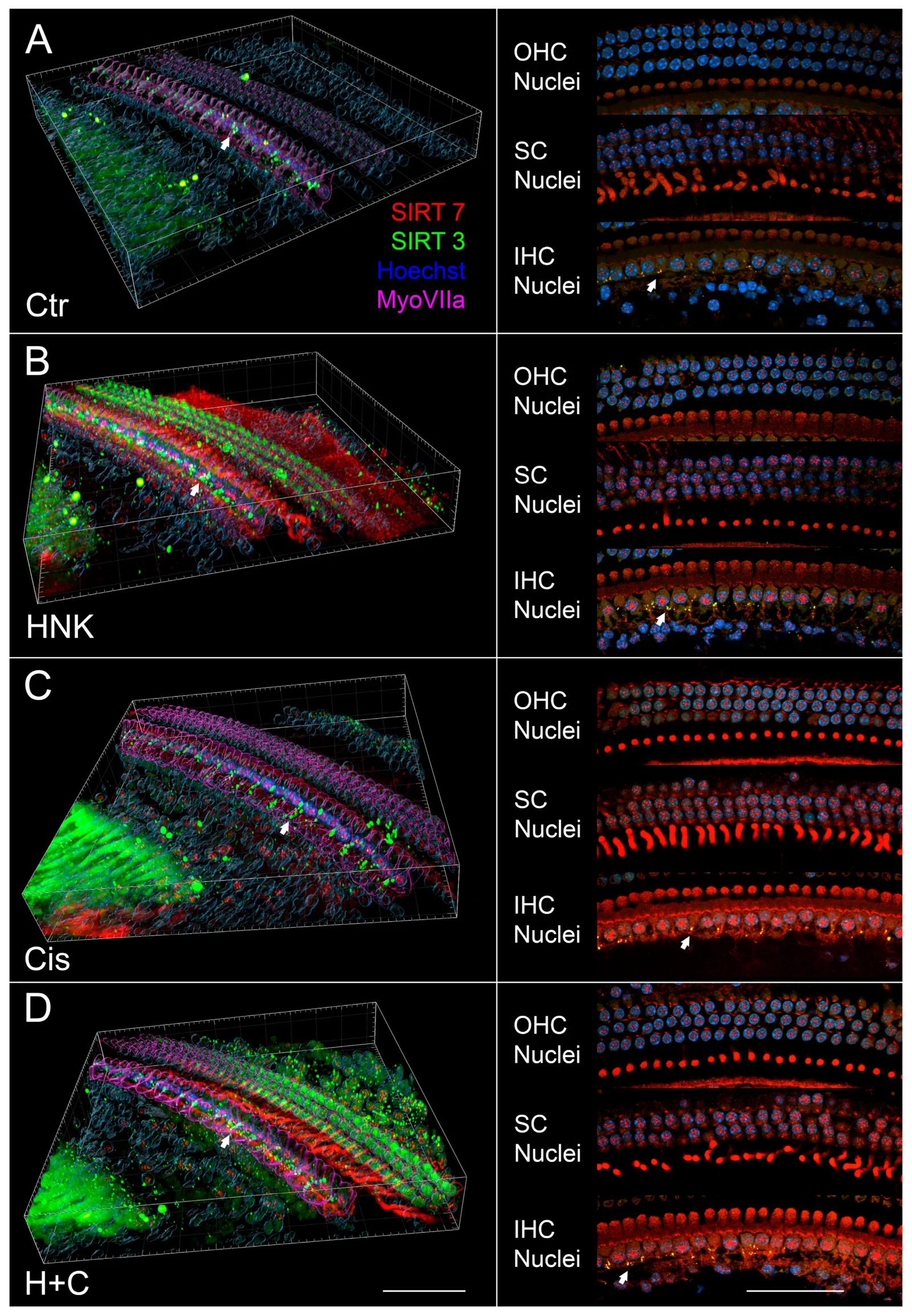
Nuclear Sirt7 expression and colocalization with Sirt3 in the cochlea (frequency range: 9.5–19.1 kHz) after different treatments. (**A**) Control; (**B**) HNK10-only; (**C**) Cis-only; and (**D**) HNK10+Cis. The left panels show the comprehensive expression patterns of Sirt3 and Sirt7 in the 3D views of confocal image stacks. The right panels show Sirt7 expression focusing on the nuclei of OHCs, IHCs, and SCs. Sirt3 was used to confirm the expression pattern in the cochlea and upregulation by honokiol (the left panels). Sirt7 signal was observed in the nuclei in a granular pattern and was upregulated by both honokiol and cisplatin (the right panels). White arrows show puncta of Sirt3 at the bottom regions of IHCs. Note that Sirt3 was expressed mostly in the soma, and therefore the signal in the nuclear region in the right panels was relatively weak. Scale bars: 50 µm.

Figure 7 shows the expression of Sirt7, one of the nuclear sirtuins, in the cochlea, using Sirt3 as a positive control for confirming honokiol efficacy. Consistent with the previous results, the Sirt3 signal (green) was upregulated by honokiol, as evident in the 3D views in the left panels of Figure 7A–D. The bright puncta of Sirt3 were again observed in specific locations close to the bottom of the IHCs, as indicated by the white arrows. Conversely, the Sirt7 signal (red) exhibited a distinct granular pattern in the nuclei. The right panels in Figure 7 showed Sirt7 expression in the nuclei of OHCs, IHCs, and SCs, which was notably upregulated by both honokiol and cisplatin treatment. Comprehensive expression patterns and colocalization of different fluorescence signals were shown in Figure S6. Pronounced Sirt7 expression was also detected in pillar cells, especially after cisplatin and honokiol treatment (Figure S6, the yz orthogonal views).

#### 2.4.3. Upregulation of Cytoplasmic Sirtuins by Honokiol

Sirt1 and Sirt2 are the two cytoplasmic sirtuins; the expression of Sirt1 and Sirt2 in the cochlea was also studied. Given that bright puncta expression at the basal pole of the IHCs close to the ribbon synapse region was a recurring observation across multiple sirtuins (Sirt1, 2, 3, and 5) in our pilot studies and as shown in Figure 6 and Figure 7, GluR2 (green) was employed as a postsynaptic marker to investigate the potential relationship between these sirtuin aggregations and ribbon synapses. Comprehensive confocal imaging of Sirt1 expression was shown in Figure S7. Sirt1 fluorescence signal (red) was observed in the cytoplasm of the hair cells (Figure S7A), which was upregulated by honokiol (Figure S7B,D) while potentially suppressed by cisplatin (Figure S7C). No obvious upregulation was observed in the SCs, no matter whether the samples were treated with honokiol or cisplatin. Furthermore, bright Sirt1 puncta were observed at the bottom of IHCs and were partially co-localized with GluR2, as indicated by the white arrows and the 3D projection plots in Figure S8. In addition, Sirt2 expression shared a highly similar pattern to Sirt1 in one batch of immunostaining, as shown in Figure S9.

**Figure 8:**
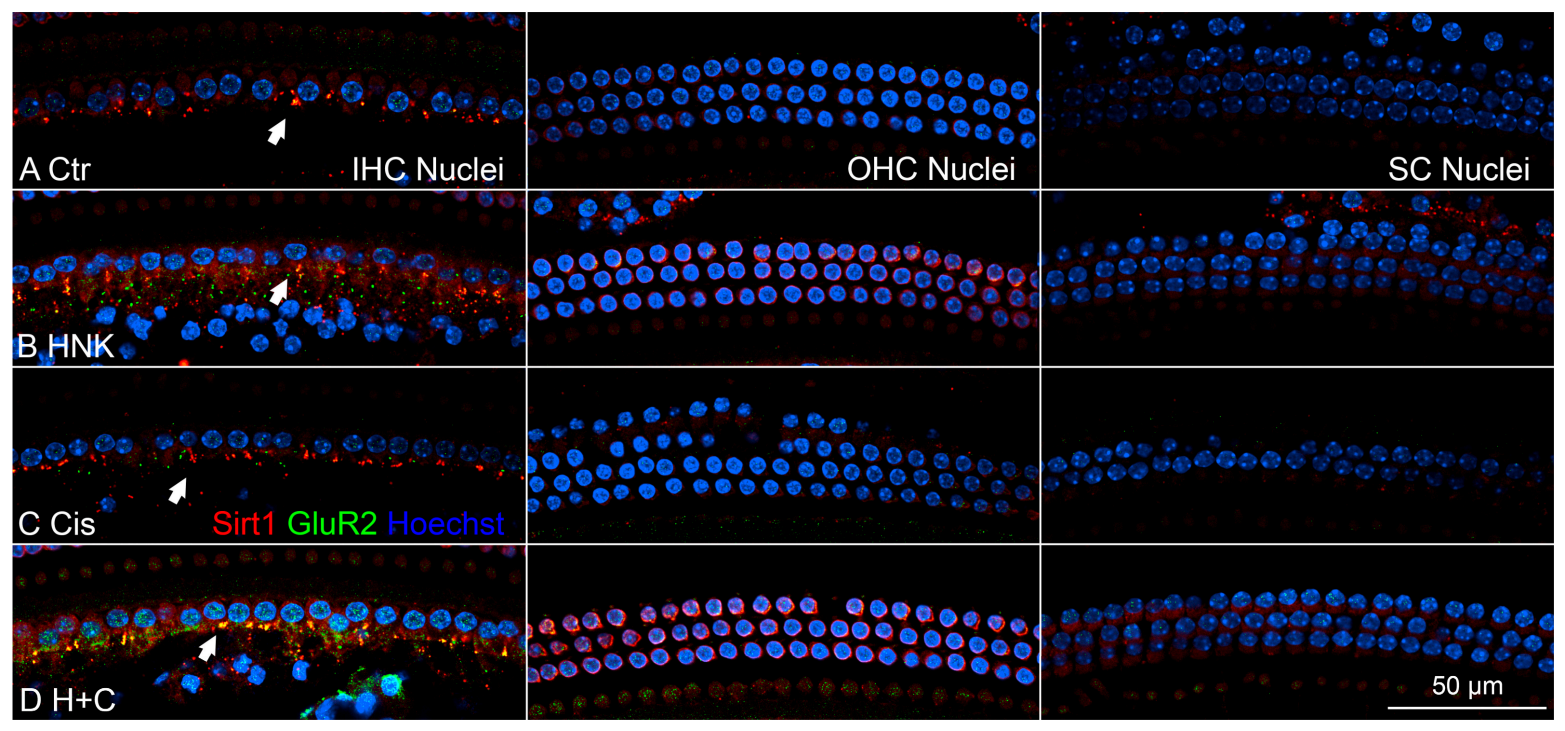
Cytoplasmic Sirt1 and postsynaptic GluR2 expression in the nuclear regions of the cochlea (frequency range: 19.1–36.5 kHz) after different treatments. (**A**) Control; (**B**) HNK10-only; (**C**) Cis-only; and (**D**) HNK10+Cis. The left, central, and right panels focused on the nuclei of IHCs, OHCs, and SCs, respectively. Sirt1 (red) expression was detected in hair cells and upregulated following honokiol treatment (**B**,**D**). Note that no nuclear localization was observed for Sirt1, although upregulated expression in the peri-nuclear area was observed after honokiol treatment. White arrows indicate Sirt1 puncta observed at IHC synaptic regions, which were partially colocalized with the postsynaptic marker GluR2 (green).

Given that Sirt1 and Sirt2 have been reported to exhibit nuclear localization in mammals, we conducted a close examination of Sirt1 expression in the nuclei of the hair cells and SCs. As shown in Figure 8, no definitive Sirt1 fluorescence signal was detected within the nuclei of IHCs, OHCs, or SCs. In contrast, a robust Sirt1 signal was observed in the peri-nuclear area of the OHCs after honokiol treatment (Figure 8B,D, the central panels). Again, puncta of Sirt1 were clearly observed in the vicinity of the IHC nuclear area, partially colocalized with GluR2 (Figure 8, left panels; also refer to Figure S7, the surface and orthogonal views focusing on synapses, as well as Figure S8). The exact component and function of these puncta are not clear at this point, although it is highly possible that they are associated with ribbon synapses as indicated by the co-localization with GluR2.

### 2.5. Transcriptional Upregulation of Sirt3 by Honokiol

**Figure 9:**
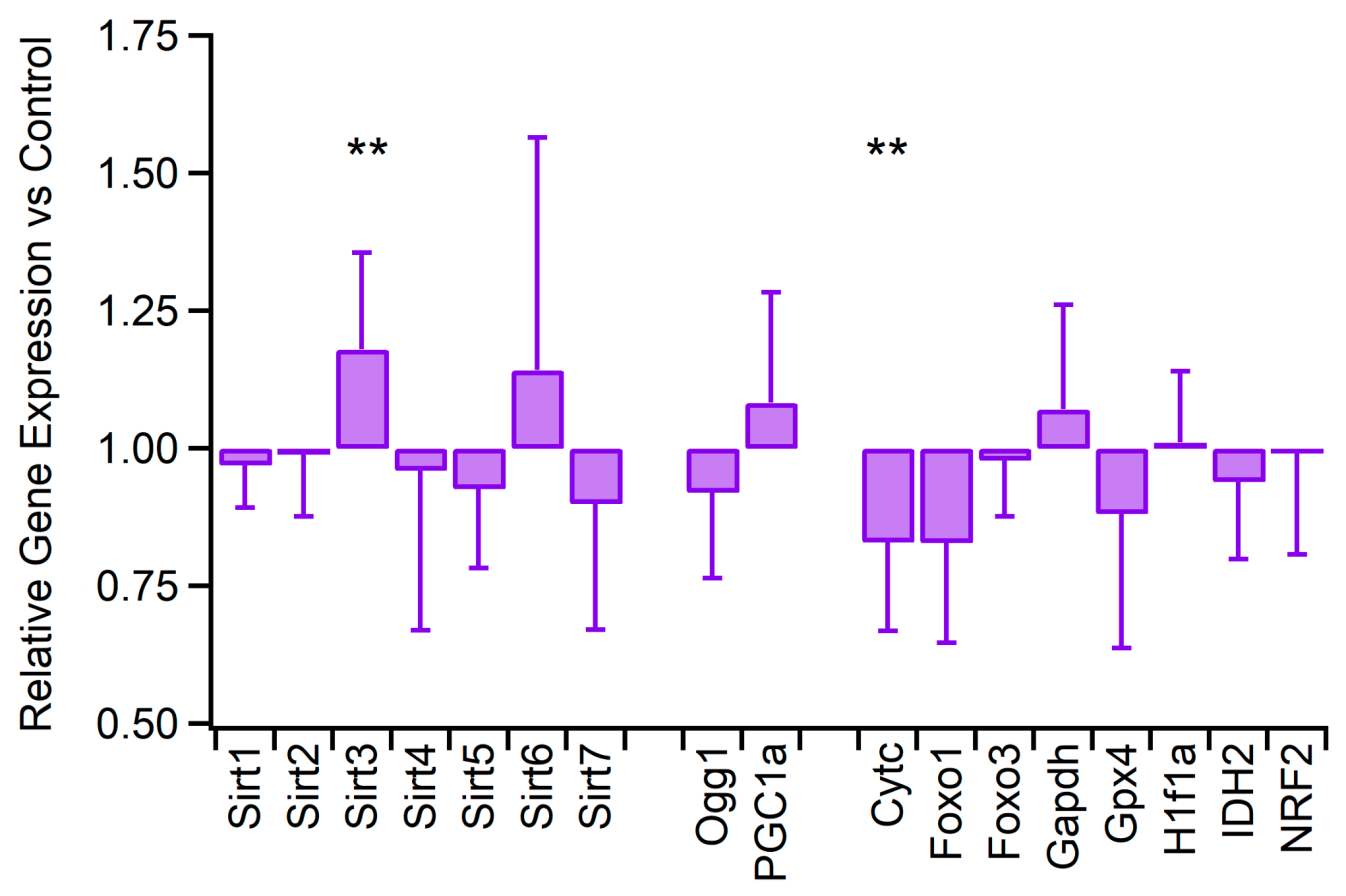
RT-qPCR showing gene expression profile in the cochlea of honokiol-treated mice. Relative expression (Y-axis) was calculated using the 2^−∆∆Ct^ method, normalized to Actb. Bars indicate fold changes in gene expression in the honokiol-treated group versus vehicle controls. Statistical significance was calculated for each gene by an unpaired two-tailed Student’s *t*-test comparing the HNK10-only group (*n* = 4) versus the control group (*n* = 3). Error bars represent standard deviation. ** *p* < 0.01.

To further corroborate honokiol-induced sirtuin upregulation observed at the protein level, RT-qPCR was performed to assess the relative mRNA expression of sirtuins and associated genes in the cochlea. Mice in the HNK10-only and Ctr groups were used, and cochleae were also harvested on Day 5 after four doses of treatment. Figure 9 shows the transcriptional profile in the form of relative expression changes calculated using the 2^−∆∆Ct^ method. Within the sirtuin family, honokiol treatment induced a significant upregulation of Sirt3 mRNA expression (1.32 ± 0.08-fold change relative to control, *p* = 0.0007). The transcript levels of the other sirtuins remained unaffected by the treatment.

To map the molecular pathways driving and executing sirtuin upregulation, we evaluated a panel of genes spanning the sirtuin regulatory network. This included two critical upstream regulators: peroxisome proliferator-activated receptor gamma coactivator 1-alpha (PGC-1α), the master regulator of mitochondrial biogenesis targeting Sirt3 [37,60,61,62], and 8-oxoguanine DNA glycosylase (OGG1), an enzyme essential for transcriptional upregulation of Sirt1 [63]. Additionally, eight downstream targets related to sirtuin signaling were investigated to assess pathways involved in apoptosis (Cytochrome c, Cytc; Forkhead box O1 and O3, Foxo1 and Foxo3), as well as redox homeostasis and stress response (Glyceraldehyde 3-phosphate dehydrogenase, Gapdh; Glutathione peroxidase 4, Gpx4; Hypoxia-inducible factor 1-alpha, Hif1α; Isocitrate dehydrogenase, Idh2; Nrf2). Notably, among these associated pathways, only the transcript level of Cytc was significantly downregulated (0.70 ± 0.15-fold change, *p* = 0.0004) following honokiol administration. These results suggest that, while modest changes were observed in mRNA expression of Sirt3 and Cytc, honokiol-mediated protection of cochlear function is potentially mediated through post-translational deacetylation or other transcriptional regulations.

## 3. Discussion

This study verified the hearing-protective effects of honokiol in a three-cycle cisplatin treatment regimen simulating chemotherapy in a tumor-bearing mouse model. Pre-treatment with honokiol prevents cisplatin-induced ABR threshold shift and synaptic loss while protecting the general health of the animals during cisplatin treatment. Meanwhile, sirtuin family members in the mitochondria (Sirt3 and Sirt5), cytoplasm (Sirt1 and Sirt2), and nuclei (Sirt7) were all upregulated by honokiol treatment at the protein level. Among them, Sirt3 upregulation was confirmed at the mRNA level. These results strongly support our hypothesis that sirtuins are upregulated during honokiol-mediated hearing protection against cisplatin ototoxicity, although sirtuin-associated regulatory transcripts tested in this study largely remained stable in honokiol treatment.

CIHL is a severe sequela of chemotherapy, which is notoriously difficult to address due to various challenges. Interference with the therapeutic effects of cisplatin is the biggest concern, as hearing-protective candidates may deactivate cisplatin [17,18,19] or protect cancer cells [20]. Local [18,64,65,66] or delayed [67,68] drug administration is often required for these candidates. To date, sodium thiosulfate is the only FDA-approved treatment for CIHL, with restricted application time and limited use in pediatric patients [21,69].

Honokiol is a plant-originated multifunctional polyphenol [39] with tumor suppression function and protective effects against various stress or toxicity in multiple tissues and organs, as reviewed in our other publications [37,38]. More importantly, honokiol can enhance conventional anticancer agents; therefore, it is potentially a viable adjunct in combination therapies with cisplatin [47]. It has been shown that honokiol is synergistic with cisplatin in multiple cancer models, including colon cancer [45], ovarian cancer [70], and oral squamous cell carcinoma [71]. Mechanistically, Sirt3 activation seems to play critical roles [42,72,73], and honokiol can also sensitize the cisplatin-resistant phenotype through modulating IL-6/Stat3 signaling [71], a critical pathway involved in tumorigenesis and development [74]. It has also been shown that honokiol mitigates severe systemic toxicities, such as acute kidney injury, induced by cisplatin [40,75,76]. A recent review has summarized the application of honokiol in combination therapy for cancer treatment [47].

Our group further demonstrated that honokiol protects against CIHL in cell cultures and in wild-type and tumor-bearing mice [38] (Figure 2). This honokiol-mediated hearing protection is shown at the ABR, hair cell, and synaptic levels [38] (Figure 4), as well as in an early-onset hearing loss model [48]. Furthermore, honokiol brings additional benefits such as enhanced tumor suppression, improved survival rate, and mitigated weight loss. While the high dose of honokiol (20 mg/kg/day) shows a slightly stronger hearing-protective effect than the other two doses (Figure 2D), the middle dose (10 mg/kg/day) has the best overall effect in both protecting the hearing and improving the general health of the animals (Figure 3). Mechanistically, honokiol reverses cisplatin-induced global transcriptomic downregulation in OHCs. Genes in multiple signaling pathways are involved, especially those affecting mitochondrial functions [55]. These results strongly support that honokiol is a promising candidate for treating CIHL.

In our study, the cisplatin-induced ABR threshold shift exhibited a more attenuated profile compared to those reported in previous studies using the same three-cycle regimen. While prior studies documented severe hearing loss spanning almost the entire tested frequency range (4–32 kHz), significant hearing loss was only observed at 32 kHz following the completion of the three cycles in our study. This discrepancy can potentially be attributed to two reasons. First, the criteria for the humane endpoint implemented in our protocol might be different from those in the previous studies. In our study, mice showing body weight loss exceeding 25% would be excluded from further investigation and analysis, a threshold considerably more restrictive than close to 40% weight loss permitted in other studies [54]. More severe weight loss might have reflected more severe systemic toxicity, which could further exacerbate cochlear function. Because these animals did not survive to the end of the third cycle, our reported ABR thresholds may have introduced a survival bias which underestimated the true severity of cisplatin ototoxicity. Second, premature termination of the treatment regimen due to oversized tumor growth (1500 mm^3^) might have also introduced confounding variables when evaluating the final cumulative ototoxicity. Consequently, the overall magnitude of CIHL observed in our study was relatively subtle, which inherently limits the reliability in determining honokiol-mediated hearing protection, in terms of therapeutic magnitude and frequency range.

Besides traditional antioxidants, candidates including factors/cytokines in metabolic [77,78], inflammatory [79,80], or apoptosis [52,81,82] signaling pathways were also tested more recently with promising results. Polyphenols, a diverse group of natural compounds abundant in plants with antioxidant and anti-inflammatory functions, present a novel type of candidate for hearing protection against CIHL, which might bring additional benefits (for review, see [83,84]). For example, luteolin, a flavone derived from celery, green pepper, and chamomile, has anti-inflammatory and anti-carcinogenic properties and alleviates ferroptosis in CIHL [85]. Resveratrol, a compound abundant in grapes and red wine with various protective effects, protects against CIHL in guinea pigs [86] and rats [87]. Curcumin, a food supplement with chemoprotective, antioxidant, and anti-inflammatory activities, also protects against CIHL in mice and rats [88,89]. Nevertheless, whether these polyphenols interfere with the therapeutic effects of cisplatin is yet to be determined. In addition, bioavailability might be another concern, since most of these polyphenols are water-insoluble, including honokiol. Special formulations improving the solubility, such as liposomes and amorphous solid dispersions, might help for their clinical applications.

ROS plays a central role in triggering the inflammatory and apoptotic pathways in CIHL [90] as well as in other types of hearing impairment [e.g., noise-induced hearing loss (NIHL) [91,92]; drug-induced hearing loss [11,93,94]; age-related hearing loss (ARHL) [95,96,97]; for a recent review, see [98]]. Following cisplatin exposure, ROS are generated at multiple sites along interconnected signaling pathways, such as inflammation and apoptosis, thereby causing damage across different cellular compartments [17,99,100]. Consequently, targeting a single site or pathway of ROS generation might be insufficient to achieve comprehensive protection against CIHL.

The sirtuin family comprises seven highly conserved members [22,23,24] that are distributed across different cellular compartments and may, therefore, form an intrinsic anti-ROS network through regulating distinct downstream substrates [23,25,26,27]. Sirtuins play critical roles in metabolism [28], longevity [25,29], and carcinogenesis [29] by governing multiple cellular processes including homeostasis [101], responses to stress [102], DNA damage repair [102], inflammation [103,104], and apoptosis [103]. However, the functions of individual sirtuins and their mechanisms in hearing protection remain largely unknown. A previous study examined cochlear sirtuin expression and age-related changes in cochlear sections [36]. However, the results require further verification due to limitations in immunostaining quality and the unclear pattern of sirtuin expression in different cellular compartments (such as cytoplasm versus nuclei). In the present study, we found that multiple sirtuins were upregulated in honokiol-mediated hearing protection against CIHL, suggesting their potential roles in this process. In addition, this upregulation was more evident at the protein level than at the mRNA level.

Individual sirtuins may have different functions in different processes. In hearing protection, studies have indicated the significance of Sirt1–3 in hearing loss and protection. Sirt1 has been shown to protect cochlear hair cells [105] and slows ARHL [106,107], associated with P53 [105], through regulating cellular processes such as autophagy [107]. In contrast, hemizygous Sirt1 knockout mice with a deficiency of Sirt1 prevented ARHL that normally occurred in wild-type animals [108]. Noise exposure upregulates Sirt2 expression, and treatment with a Sirt2 inhibitor reduced NIHL [109], indicating a negative role of Sirt2 in the protection of cochlear function. Nevertheless, Sirt2 upregulation is a protective mechanism in other tissues [110,111]. Sirt3 is involved in multiple intra-mitochondrial metabolic processes, including energy production, ROS generation, and detoxification [112]. The involvement of Sirt3 in hearing protection against different insults, including noise [113,114], drugs [115,116], and age [95,96] has also been studied. Glycolytic metabolism seems to play critical roles in Sirt3-dependent hearing protection, as demonstrated in an acute CIHL model [116]. Various compounds and treatments have been tested for hearing protection through Sirt3 activation in these studies, although none of the tested candidates are suitable for clinical application against CIHL [38]. In our current study, although Sirt5 and Sirt7 were also upregulated during honokiol-mediated hearing protection, their function and influence are yet to be studied.

As a reported Sirt3 activator, the underlying mechanisms of honokiol are believed to be largely associated with Sirt3 [42], as shown in the heart [117,118], brain [119,120], and kidney [121]. Our recent transcriptomic study revealed a broad upregulation of antioxidant genes by honokiol [55]. Results in the current study further confirmed that honokiol upregulated Sirt3 at the mRNA level and suggested that other sirtuins were also upregulated at the protein expression level. To gain a comprehensive understanding of honokiol-mediated cochlear protection, the upstream and downstream pathways involved in the upregulation of the sirtuin family were further investigated using RT-qPCR. Two upstream regulators for sirtuins in mitochondria (PGC-1α, for Sirt3) and cytoplasm (OGG1, for Sirt1) that are essential for mitochondrial function and cisplatin damage were tested, although none were significantly altered. Other tested downstream effectors are all critically involved in multiple cellular processes as abovementioned. These include direct targets of Sirt3 (FoxO3 and Idh2) and Sirt1 (Nrf2) that upregulate intrinsic antioxidant defense. Unfortunately, the only observed significant change was the decrease in the downstream gene Cytc, an indicator of the mitochondrial-dependent apoptosis pathway, after honokiol treatment. On the other hand, Hif-1α, a stress-responsive transcription factor that is suppressed by Sirt3-mediated ROS reduction, is unchanged with honokiol treatment. Although more direct evidence for the association between sirtuin upregulation and hearing protection at the molecular level is missing, our studies provided new insight into the strategy for treating CIHL. That is, comprehensive activation of the intracellular anti-ROS system from different cellular compartments. Future work, such as transcriptomic studies and further functional verifications, will help to confirm the roles of individual sirtuins in this process.

An intriguing finding of the present study was the detection of high punctate sirtuin immunoreactivity (Sirt1, 2, 3, and 5) within the IHC ribbon synaptic region partially colocalized with postsynaptic GluR2, which is also potentially upregulated by honokiol. This is the first time that sirtuin expression in the ribbon’s synaptic region is reported. Although the specific localization and functions of these sirtuins remain largely unknown, evidence from other neuronal systems might give some insights. Sirt3 is found in the mitochondria of presynaptic terminals, where it supports ATP production and thereby sustains synaptic transmission [122]. Sirt2 is enriched at the synapses of hippocampal neurons, regulating the trafficking, stability, transmission, and plasticity of GluA1, another subunit of the same AMPA receptor family as GluR2, through deacetylation [123]. Other studies have also shown potential protection of ribbon synapses by Sirt1 [124] and Sirt3 [125,126] while missing solid data on their synaptic localization. Although further studies are needed to confirm the roles of sirtuins in ribbon synapses and sirtuin-dependent or independent mechanisms underlying honokiol protection, these observations strongly suggest that sirtuins might be important for maintaining and protecting synaptic functions.

Despite the promising findings of the current study, there are several limitations. First, the severity of CIHL observed in our tumor-bearing mouse model was relatively milder, as discussed above, which inherently limits the reliability of honokiol-mediated hearing protection. Second, although a clear correlation between honokiol administration and the upregulation of various sirtuin family members was shown, a direct causal connection between sirtuin upregulation and hearing protection is currently missing. Follow-up studies, such as sirtuin-specific knockouts or pharmacological inhibition, are necessary to definitively confirm the mechanistic roles of individual sirtuins in CIHL. Third, immunofluorescence intensity can be affected by various factors such as antibody affinity, tissue penetration, and photobleaching. Therefore, sirtuin expression and upregulation evaluated with fluorescence intensity should only be considered quasi-quantitative and interpreted as relative changes rather than quantitative changes in protein concentration. Upregulation in sirtuin protein levels does not necessarily indicate increased enzymatic activity, either. Furthermore, more tumor models are necessary to verify honokiol-mediated hearing protection against CIHL due to the diverse oncology of different tumor models.

## 4. Materials and Methods

### 4.1. Ethics Statement

All procedures were carried out following the NIH Guide for the Care and Use of Laboratory Animals and approved by the Institutional Animal Care and Use Committee at Northwestern University, Chicago, USA (protocol number IS00018749).

### 4.2. Animal Groups and Treatments

MMTV-PyMT mice (Jackson Laboratory, Bar Harbor, ME, USA, Jax Stock #: 002374, FVB background) were used in this study as a breast tumor model. The MMTV-PyMT is an oncogenic RNA virus of the *Retroviridae* family, which drives breast tumorigenesis once activated. Tumor growth was monitored using a caliper when tumors were palpable. Tumor size (volume) was calculated using the formula [38,127]: (length × width^2^)/2. If multiple tumors were developed, their volumes were added to the total tumor size of the animal. Female MMTV-PyMT carriers develop palpable tumors reaching a total size of 500 mm^3^, the criteria for intervention, at 70.8 ± 10.3 days of age (*n* = 90).

Once the total tumor size reached 500 mm^3^, mice were randomly assigned to one of seven treatment groups detailed below (Table 1). A chemotherapy regimen was then performed consisting of three cycles [38,50,52]. Each cycle included four consecutive days of drug administration, followed by a 10-day recovery interval (Figure 1) [38,51]. Therefore, each cycle consisted of 14 days, and the entire regimen lasted for 42 days. Honokiol and cisplatin were both purchased from MilliporeSigma (Burlington, MA, USA) or Tocris Bioscience (Minneapolis, MN, USA). Honokiol (98% purity) was dissolved in corn oil (MilliporeSigma) and cisplatin in saline. Both were administered via intraperitoneal (i.p.) injection, with honokiol (or corn oil) given one hour prior to cisplatin (or saline). Hydration with Ringer’s Lactated Solution (RLS) was provided daily at 25 µL/g for the first seven days of each cycle.

Honokiol [76,117,128] and cisplatin [38,51,52] doses were determined based on published studies. Following systemic administration, honokiol reaches peak plasma concentration at approximately 30 min [129]. A one-hour interval was adopted to ensure optimal cochlear tissue saturation and activation of intracellular signaling pathways. Human equivalent dose of honokiol, calculated using the standard body-surface-area conversion described in FDA guidance, was approximately 0.41–1.62 mg/kg for the doses used in this study (5–20 mg/kg).

Animals were monitored daily for side effects of cisplatin treatment throughout the study. Treatment for pain will be performed when the animals show signs such as reduced grooming, reduced level of spontaneous activity, piloerection, and hunched posture. The tumor growth was measured every 2 days, and blood creatinine level was measured once per week. The animals meeting any of the following criteria were removed from the study and euthanized: weight loss > 25%, total tumor size > 1500 mm^3^, tumor diameter over 20 mm along any axis (IACUC predetermined limit), tumor burden visibly affecting the host, creatinine blood level above 2.72 mg/dL, or other physical ailments detrimental to long-term wellbeing. Among them, oversized tumor burden mainly occurred in non-cisplatin-treated groups, and animal loss or excessive weight loss was mainly observed in cisplatin-treated groups. After the third cycle, all surviving animals were euthanized for histology.

In addition, female wild-type FVB mice (Jackson Laboratory, Jax Stock #: 001800) treated with honokiol at a dose of 20 mg/kg/day (*n* = 8) or a vehicle (*n* = 6) were used as the healthy control. To show sirtuin expression, another 20 animals were randomly assigned to the four groups (5 in each group) but were treated for only four doses and euthanized on Day 5. These mice are also listed in Table 1.

**Table 1:** Animal groups and treatments.

Strains	Group #	Group Name	Number	Treatment
MMTV-PyMT female carrier	1	Control	*n* = 19	corn oil + saline (vehicle only)
	2	Cis-Only	*n* = 21	corn oil + cisplatin (4 mg/kg/day)
	3	HNK10-Only	*n* = 9	honokiol (10 mg/kg/day) + saline
	4	HNK5+Cis	*n* = 11	honokiol (5 mg/kg/day) + cisplatin (4 mg/kg/day)
	5	HNK10+Cis	*n* = 12	honokiol (10 mg/kg/day) + cisplatin (4 mg/kg/day)
	6	HNK20+Cis	*n* = 12	honokiol (20 mg/kg/day) + cisplatin (4 mg/kg/day)
Female FVB wild type *	1	Control	*n* = 6	corn oil + saline (vehicle only)
	2	HNK20-Only	*n* = 8	honokiol (20 mg/kg/day) + saline
MMTV-PyMT female carrier **	1	Control	*n* = 5	corn oil + saline (vehicle only)
	2	Cis-Only	*n* = 5	corn oil + cisplatin (4 mg/kg/day)
	3	HNK10-Only	*n* = 5	honokiol (10 mg/kg/day) + saline
	4	HNK10+Cis	*n* = 5	honokiol (10 mg/kg/day) + cisplatin (4 mg/kg/day)

* for ABR controls. ** for immunostaining, treated for one cycle only and euthanized on Day 5.

### 4.3. ABR Measurement and Data Processing

The cochlear function was evaluated by ABRs, which were recorded before (Day 0) and after the treatment was initiated (Days 14, 28, and 42). The detailed method for ABR measurement has been described in our previous publications [38,130]. Briefly, animals were anesthetized by a mixture of ketamine (80–100 mg/kg) and xylazine (2–5 mg/kg), diluted at 1:20 in RLS. The level of anesthesia was monitored every 15 min using the paw withdrawal reflex and maintained with ketamine dilution (1:20 in RLS). The animals’ body temperature was maintained at 37 °C using a water-filled heating pad and monitored every 15 min along with other vital signs (O_2_ saturation, respiratory rate, and pulse rate).

A customized system and a Tucker-Davis Technologies (TDT, Alachua, FL, USA) system were used for ABR recordings. For the customized system, signal generation and data acquisition were controlled by custom-written software in Testpoint 7.1 (SuperLogics, Inc., Massachusetts, MA, USA). The sampling rate was set at 250 kHz. Pure tone bursts (36, 32, 24, 16, and 8 kHz) were generated with a computer I/O board (KCPI 3110, Keithley, Cleveland, OH, USA), attenuated in 5 dB steps, amplified, and delivered via a Beyer DT770Pro speaker pre-calibrated with a Brüel and Kjær 1/8-inch microphone (beyerdynamic GmbH & Co. KG, Heilbronn, Germany). Signals were delivered via a speculum positioned in the ear canal. Tone pips with a 5 ms duration, 1 ms rise/fall time, and a maximal sound level of ≤107 dB SPL (sound pressure level re 20 µPa) were adopted. To elicit ABR signals, three hypodermic needles were placed at the ipsilateral mastoid (high), vertex (low), and the body (common ground). The electrodes were connected to an ISO-80 differential amplifier (World Precision Instruments, Sarasota, FL, USA) with an 80 dB amplification and a band filter set between 300 and 3 kHz (slope: −12 dB/octave). A frequency device filter (Hewlett Packard, Palo Alto, CA, USA) with a slope of −48 dB/octave and a gain of 20 dB was used for further filtering and amplification. Stored signals for all frequencies and sound levels were averaged 256 times or until the signal/noise ratio ≥4.

For the TDT system, an RZ6 processor was used for generating pure-tone pips, and a Medusa4Z was used to drive a pre-calibrated speaker. ABR signals were recorded using a WS4 Computer Workstation. BioSigRZ software (Version 5.7.6) was used to display and acquire data. The attenuation was 21 dB, and the gain was 0 dB. The sound level was set between 85 and 20 dB SPL, with a step of 5 dB. The rest of the parameters and the recording electrode placement were kept consistent with the customized system.

ABR threshold was defined as the minimum level required for an ABR with a root mean square (RMS) 1.5 times above the noise floor. Cumulative plots were generated by first rank ordering the ABR thresholds for each frequency and each group and then binning the thresholds with a bin size of 5 dB (Figure 2 and Figure S2) [53].

### 4.4. Animal Euthanasia, Cochleae Harvest, and Cochlear Dissection

At the end of the treatment, the animals were euthanized by Euthasol^©^ injection (Virbac AH, Inc., Fort Worth, TX, USA, 0.1 mL, i.p.), followed by cardiac perfusion with 4% paraformaldehyde (PFA). Cochleae were then harvested, followed by in-cochlea PFA injection, and left in PFA for 2 h for post-fixation. Decalcification was then performed using 10% EDTA (Sigma-Aldrich, St. Louis, MO, USA) in 1X PBS (diluted from 10X PBS, Invitrogen, Carlsbad, CA, USA; Cat. No. AM9624) for 2–3 days. The full-length cochlear coil was then dissected out in 1X PBS and was cut at specific locations into four segments (S1–S4). Since the apical and basal parts tend to gain artificial damage during the processing of the samples, only the middle turn of the cochlea was used for immunostaining (S2 and S3, frequency range of ~9.5–19.1 and 19.1–36.5, respectively) for better immunostaining and mounting. The frequency range was determined previously by measuring the full length of the cochlea, which was correlated with the published frequency map in CBA/J mice [38,131].

### 4.5. Immunostaining and Confocal Imaging

Standard immunostaining protocols as described in our previous publications were applied [38,114]. Briefly, cochlear samples were first soaked in 30% sucrose, frozen on dry ice, and thawed at room temperature. After washing three times with 1X PBS and rinsing for 20 min on a shaker, the samples were blocked with 5% serum + 1% Triton. Primary antibody mixes were then added, and the samples were incubated at 37 °C overnight. Depending on the purpose, the primary antibody mix contained different primary antibodies. For synapses, it had rabbit anti-Myosin VIIa (for hair cells, Proteus Biosciences, Waltham, MA, USA, 1:200), mouse (IgG1) anti-CtBP2 (for pre-synaptic component, BD Transduction Labs, San Jose, CA, USA, 1:200), and mouse (IgG2a) anti-GluR2 (for post-synaptic component, MilliporeSigma, Burlington, MA, USA, 1:500). For sirtuin family members, the antibodies for CtBP2 and GluR2 were replaced by 1–2 sirtuin family members, including Sirt1, 2, 3, and 5 (from Santa Cruz Biotechnology, Dallas, TX, USA, or Novus Biologicals, Centennial, CO, USA). After being rinsed three times in PBS (10 min each), the secondary antibody mix consisting of duck anti-rabbit (IgGH-L) AF647 (Jackson Immuno Research Laboratories, Inc., West Grove, PA, USA, 1:200), goat anti-mouse (IgG1) AF568 (Thermo Fisher Scientific, Waltham, MA, USA, 1:200), goat anti-mouse (IgG2a) AF488 (Thermo Fisher, 1:200), and Hoechst (Thermo Fisher, 1:1000), was added and incubated in the dark for 2 h at 37 °C. Primary antibodies from Santa Cruz Biotechnology are conjugated with fluorophores (Alexa Fluor 488, 546, or 594); therefore, no secondary antibody was needed for these primary antibodies. The samples will then be washed three more times with 1X PBS and mounted on slides. Three or more replicates of immunostaining were performed for all the images included in the manuscript except Sirt2.

Confocal imaging was performed on a Nikon W1 Dual CAM Spinning Disk confocal microscope (Nikon Corporation, Tokyo, Japan) at the Center for Advanced Microscopy/Nikon Imaging Center of Northwestern University. Confocal images were processed using FIJI (NIH Image) and Imaris (Oxford Instruments, Abingdon, UK) afterward. Parameters for excitation, emission, and image processing settings were fixed for all the panels in the same images presented in the paper. As an example, fluorescence intensity analysis as shown in Figure 6 was performed in FIJI by drawing a line across the desired location using the “Straight” tool, and doing “Profile Plot” under the drop-down menu “Analysis”.

### 4.6. Two-Step RT-qPCR

Mice were euthanized and cochleae extracted after four doses of honokiol or vehicle treatment. For the tissues below the round and oval windows, the incus, stapes, and nerve fibers were removed, leaving only the cochlea and cochlear bones. Kimble pestles were used to homogenize the tissues in an RNA shield at ambient temperature. The tissues were immediately centrifuged, and RNA was extracted using the Quick-RNA MiniPrep Plus Kit (Fisher Scientific, Pittsburgh, PA, USA, A46012). DNase I treatment step was performed during RNA extraction to ensure RNA purity. Extracted RNA was eluted with 50 μL nuclease-free water. Nanodrop Lite Plus was used to measure RNA concentration. The Nanodrop was blanked with nuclease-free water, and 1 μL of RNA was placed. The purity of the RNA was measured by calculating the A260/A280 ratio, with values between 1.8 and 2.2 considered pure. SuperScript IV VILO (Invitrogen, Carlsbad, CA, USA) was used for cDNA conversion, in which 300 ng of RNA was used. A previous optimization showed that 100, 300, and 1000 ng of RNA were all appropriate for the downstream qPCR. SYBR-based RT-qPCR was carried out using a QuantStudio 7 Flex Real-Time PCR system. The reaction involved 2 μL primers at 200 nM concentration, 1 μL of cDNA, 5 μL of SYBR Green master mix, and 2 μL of nuclease-free water. PCR was run for 2 min at 95 °C for enzyme activation, and 40 cycles of denaturation at 95 °C for 15 s, annealing at 60 °C for 1 min. The reaction conditions were identical for all primers used. There was a dissociation step in which the PCR was run at 95 °C for 15 s, 60 °C for 1 min, and 95 °C for 15 s. Custom primers were purchased from Sigma-Aldrich, St. Louis, MO, USA. The selection of primers was based on our recent and other publications involving Sirt3 and proteins that were involved in the Sirt3-mediated mitochondrial antioxidant pathway (e.g., [31,96,132,133]). Biological and technical replicates (4 HNK-treated mice, 3 control mice) were used. Three controls were used: the absence of RNA, reverse transcriptase, and cDNA. Three technical replicates were averaged to calculate the concentration (C_t_ values). ∆C_t_ was then calculated using the formula:

∆C_t_ = C_t_ (gene x) − CT (housekeeping gene)

∆∆C_t_ was calculated:

∆∆C_t_ = ∆C_t_ (HNK treated) − average ∆C_t_ (control)

Based on these calculations, 2^−∆∆Ct^ values were used to describe the difference in gene expression. A_ctb_ was used as a housekeeping gene.

### 4.7. Data Analysis

ABR recordings were uniformly formatted and processed using MatLab (R2023a) (The Mathworks, Inc., Natik, MA, USA), and averages and standard errors of the mean (SEM) of the thresholds were calculated and presented. One-way ANOVA (two-tailed), Rank Sum, and tests for normality were performed in Matlab for all the ABR threshold and synapse counting data. If the ANOVA indicated differences among the means, Tukey’s Honestly Significant Difference post hoc test was followed. The finalized data were plotted using IGOR Pro (Wavemetrics, Lake Oswego, OR, USA). The tests were part of a statistical package provided by IGOR Pro. Statistical decisions were made at a probability of 0.05. ABR thresholds were also analyzed using a linear mixed-effects model (Table 2). The model evaluates the fixed effects of cisplatin, treatment cycle, honokiol, and frequency (linear and quadratic components), along with their interactions. The mouse ID was included as a random intercept to account for repeated measures.

Synapse counting was initially performed in Imaris using the function of surface plot. Only the dots surrounding the bottom area of the IHCs were included, with a size range of 0.25 ± 0.3 µm, and the colocalization was recognized by the software automatically. The number of synapses was normalized by the count of IHCs in the area. Since the size of the synapses was negatively affected by cisplatin treatment, the results were verified by manual counting by three different authors based on unprocessed synaptic staining (CtPB2, GluR2, and colocalization). The reported values were based on manual counting.

## 5. Conclusions

This study demonstrated that honokiol mitigates CIHL in a tumor-bearing mouse model without compromising the therapeutic efficacy of cisplatin. Pretreatment with honokiol significantly attenuated ABR threshold shifts, loss of ribbon synapse, and health deterioration induced by cisplatin treatment. This honokiol-mediated protection is accompanied by the upregulation of multiple sirtuin family members expressed in different cellular compartments, including Sirt1 and 2 (cytoplasm), Sirt3 and 5 (mitochondria), and Sirt7 (nucleus). Puncta of multiple sirtuins (Sirt1, 2, 3, and 5) were also observed in the ribbon synapse region. RT-qPCR confirmed the upregulation of Sirt3 but not other sirtuins at the mRNA level. These findings highlight the potential of honokiol as a promising candidate for treating CIHL. Furthermore, sirtuins in different compartments form a cellular network for oxidative stress regulation. Sirtuin upregulation suggests their potential roles in honokiol-mediated hearing protection, which requires further investigation.

## Figures and Tables

**Table 2:** Linear mixed-effects model analysis of ABRs.

Fixed Effect Term	Estimate	SE	t-Value	*p* Value
(Intercept)	3.73815	0.053517	69.85	<2.2 × 10^−16^ ***
Cisplatin	0.013398	0.00847	1.58	0.1137
Cycle	0.00222	0.001364	1.63	0.1035
Honokiol	−0.005527	0.003584	−1.54	0.1231
poly(Frequency, 2)1 (linear)	3.328328	0.180299	18.46	<2.2 × 10^−16^ ***
poly(Frequency, 2)2 (quadratic)	1.488667	0.179373	8.3	<2.2 × 10^−16^ ***
Cycle:Honokiol	−0.000185	0.000072	−2.56	0.0105 *
Honokiol:poly(Fr, 2)1	−0.038165	0.014264	−2.68	0.0074 **
Honokiol:poly(Fr, 2)2	0.018241	0.014312	1.27	0.2025

* *p* < 0.05; ** *p* < 0.01; *** *p* < 0.001.

## Data Availability

The original contributions presented in the study are included in the article/Supplementary Material, further inquiries can be directed to the corresponding authors.

## Supplementary Materials

The following supporting information can be downloaded at https://www.mdpi.com/article/10.3390/ph19101618/s1. Figure S1: ABR threshold data measured in all three cycles during the chemotherapy regimen; Figure S2: Cumulative percentage plots of all the groups at 24 kHz; Figure S3: Changes in animal body weight of the different groups during the treatments; Figure S4: Tumor growth curves of the different groups during the treatments; Figure S5: Pre- (CtBP2) and post- (GluR2) synaptic changes after three cycles of treatments; Figure S6: Sirt3 and Sirt7 expressions in the cochlea (9.5–19.1 kHz) after different treatments; Figure S7: Cytoplasmic Sirt1 (red) and post-synaptic GluR2 (green) expression in the cochlea (frequency range: 19.1–36.5 kHz) after different treatments; Figure S8: Projection of confocal image stacks for Sirt1 vs. GluR2 expression in the cochlea; Figure S9: Cytoplasmic Sirt2 and post-synaptic GluR2 expression in the cochlea (frequency range: 9.5–19.1 kHz) after different treatments; Table S1. Number of animals survived after each cycle of treatment.

## Abbreviations

The following abbreviations are used in this manuscript:

ABR	Auditory brainstem response
ARHL	Age-related hearing loss
CIHL	Cisplatin-induced hearing loss
Cytc	Cytochrome c
Foxo1	Forkhead box O1
Foxo3	Forkhead box O3
Gapdh	Glyceraldehyde 3-phosphate dehydrogenase
GluR2	Glutamate receptor 2
Gpx4	Glutathione peroxidase 4
Hif1α	Hypoxia-inducible factor 1-alpha
Idh2	Isocitrate dehydrogenase
IHC	Inner hair cells
i.p.	Intraperitoneal
MMTV-PyMT	Mouse mammary tumor virus polyomavirus middle T agent oncogene
NIHL	Noise-induced hearing loss
Nrf2	Nuclear factor erythroid 2-related factor 2
OGG1	8-oxoguanine DNA glycosylase
OHC	Outer hair cell
PGC-1α	Peroxisome proliferator-activated receptor gamma coactivator 1-alpha
ROS	Reactive oxygen species
RT-qPCR	Reverse transcription quantitative polymerase chain reaction
SC	Supporting cells
SD	Standard deviations
SE	Standard error
Sirt	Sirtuin family members (e.g., Sirt1, Sirt3)
