# Supplementary material for: Sirtuin Upregulation in the Cochlea During Honokiol-Mediated Protection Against Cisplatin Ototoxicity
Source: Pharmaceuticals (Basel). 2026 Oct 12;19(10):1618. doi: 10.3390/ph19101618 (PMC13708293; doi:10.3390/ph19101618)
Supplement: Supplementary file 1 [file pharmaceuticals-19-01618-s001.zip › pharmaceuticals-4553543-supplementary.pdf]

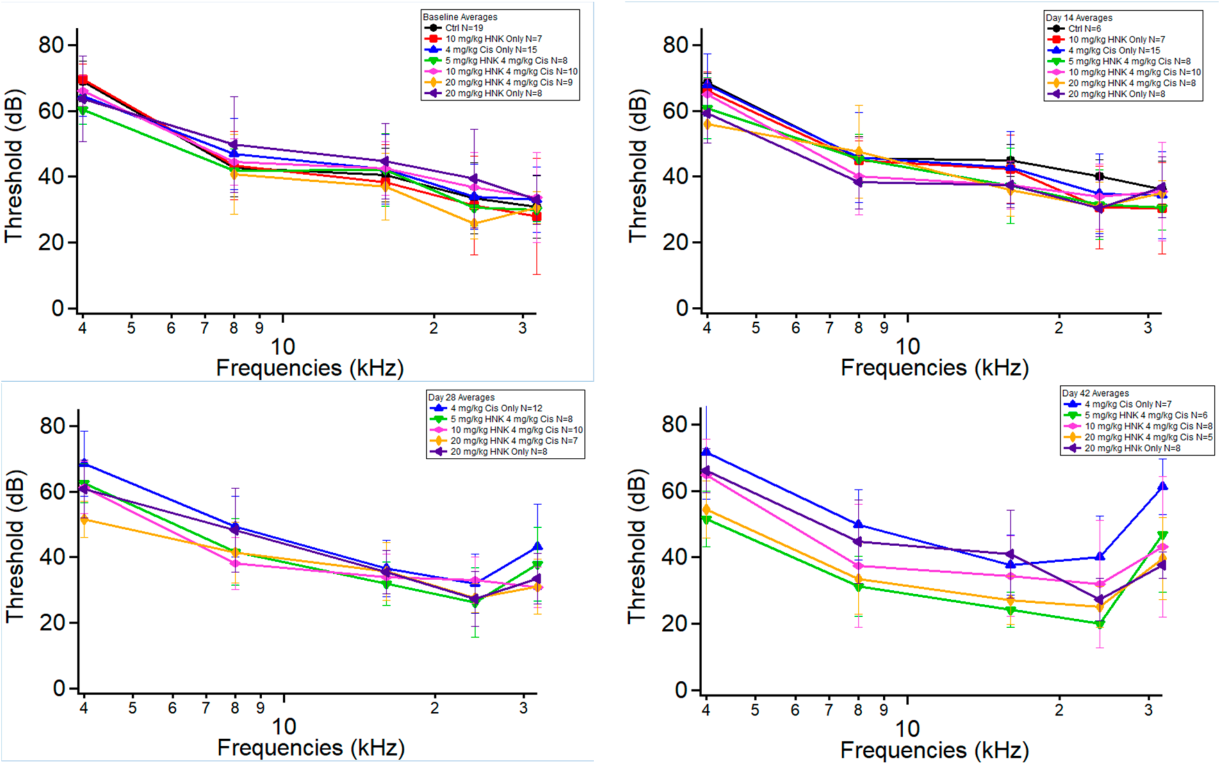

Figure S1. ABR threshold data measured in all three cycles during the chemotherapy regimen.

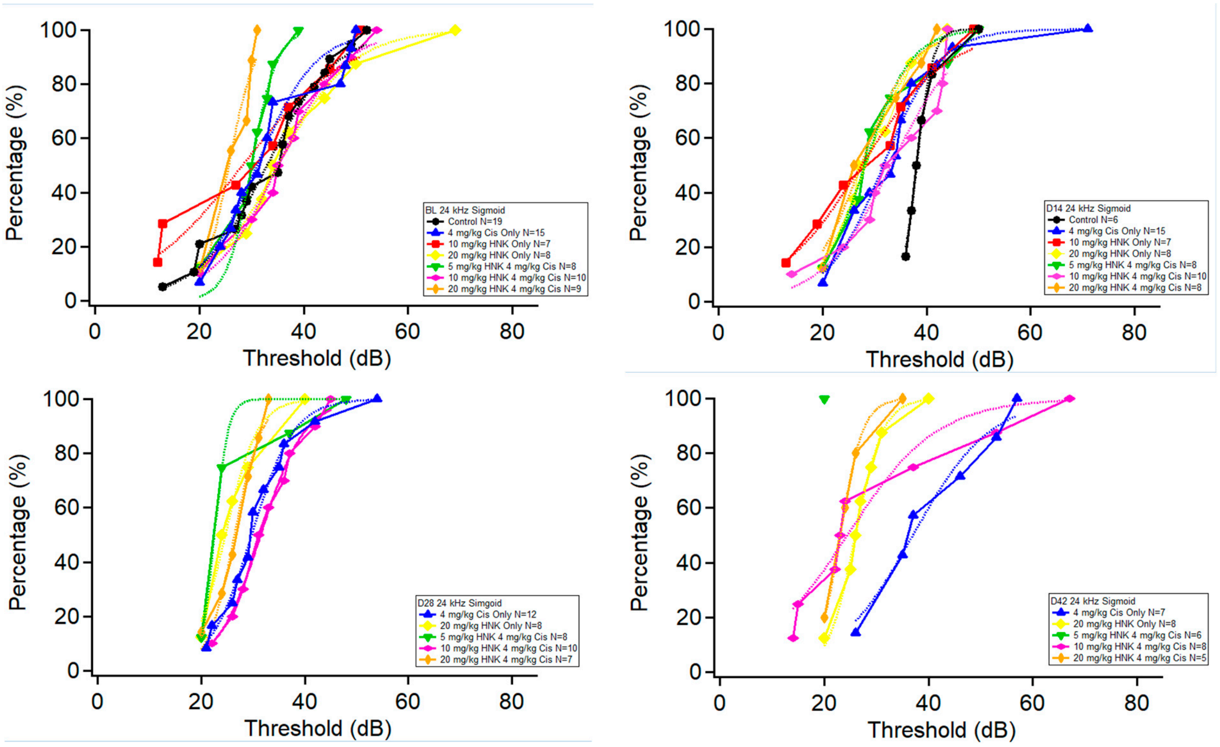

Figure S2. Cumulative percentage plots of all the groups at 24 kHz. The cumulative percentage plots of all the groups at all frequencies could be found in the attached ppx file using Igor Pro or upon request.

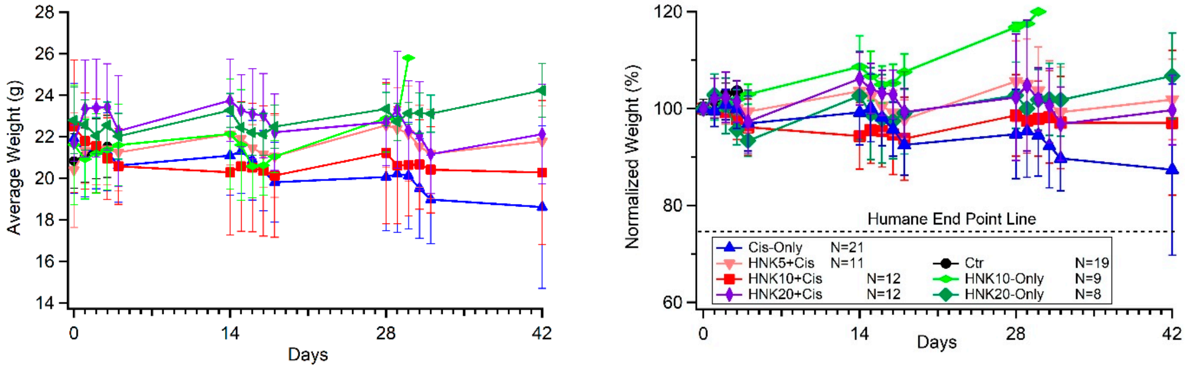

Figure S3. Changes of Animal body weight of the different groups during the treatments. (A) Average weight changes. (B) Average weight changes normalized to Day 0. The number of animals in each group was determined at the beginning of the study and gradually decreased during the treatments, which is indicated in Figure S1 and S2. Note that animals in HNK20-Only group were wild type FVB animals.

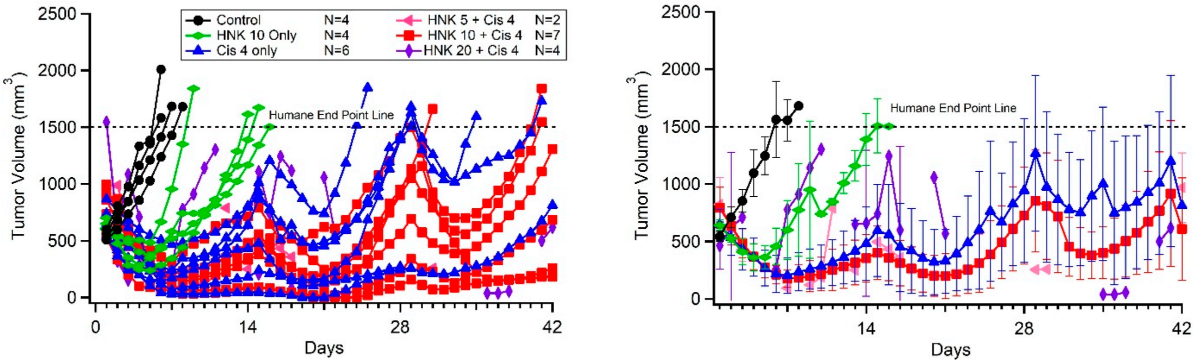

Figure S4. Tumor growth curves of the different groups during the treatments. (A) Tumor size changes in individual animals. Each curve represents one animal. (B) Averaged tumor size changes of different treatment groups. Note that the complete tumor growth was recorded for only a subset of animals in each group.

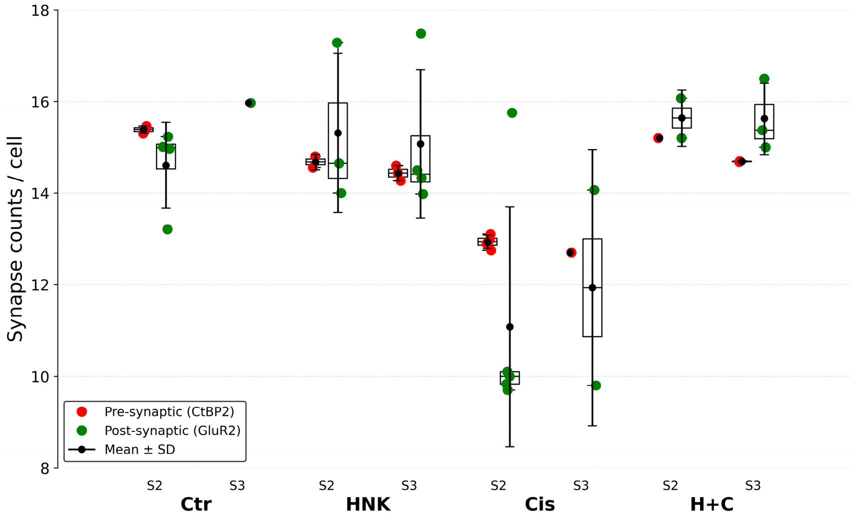

Figure S5. Pre- (CtBP2) and post- (GluR2) synaptic changes after three cycles of treatments. S2: frequency ranges of S2 and S3 are: 9.5-19.1 and 19.1-36.5 kHz, respectively. Please note that only a subset of the samples was analyzed separately for pre- and post-synaptic components.

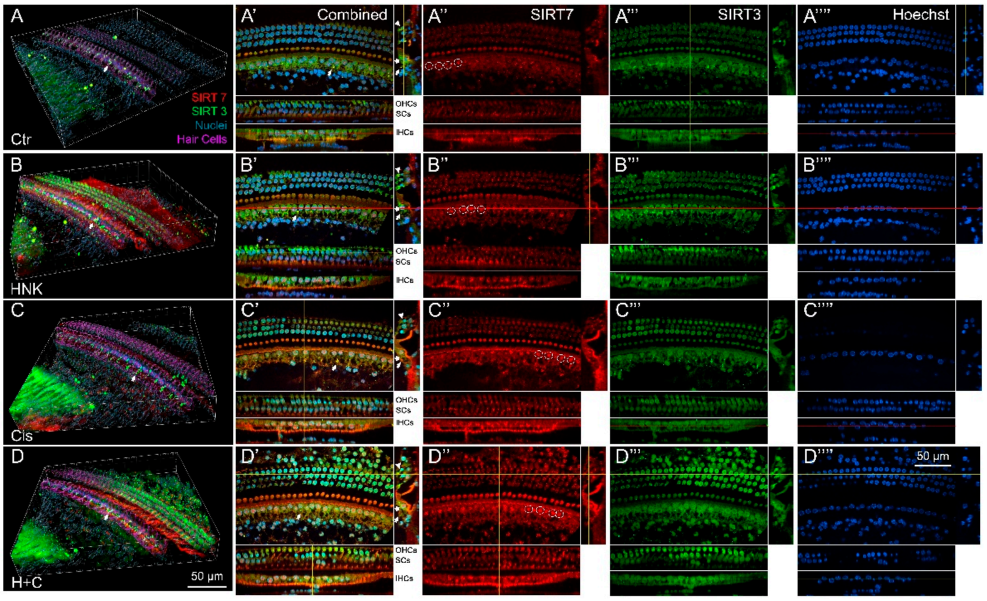

Figure S6. Sirt3 and Sirt7 expressions in the cochlea (9.5-19.1 kHz) after different treatments: (**A**) Control; (**B**) HNK10-Only; (**C**) Cis-Only; and (**D**) HNK10+Cis. The first panel of each row (**A**–**D**) shows the 3D view of confocal image stacks. The following panels (**A′**-**D′**) show combined and individual fluorescence with one surface view and three orthogonal views. The surface view focuses on the Nuclei of the IHCs. One yz orthogonal view on the right shows the entire organ of Corti, and two xz orthogonal views at the bottom focus on OHC and IHC regions, respectively. Sirt3 was used to confirm the expression in both IHCs and OHCs and upregulation by honokiol (**B** & **D**). Sirt7 signal was observed in the nuclei in a granular pattern as shown in the circles in **A′′**–**D′′** and was upregulated by both honokiol and cisplatin (**B**, **C**, & **D**). White triangles show the OHCs, white stars show the IHCs, and white arrows show puncta of Sirt3 at the IHC bottom regions.

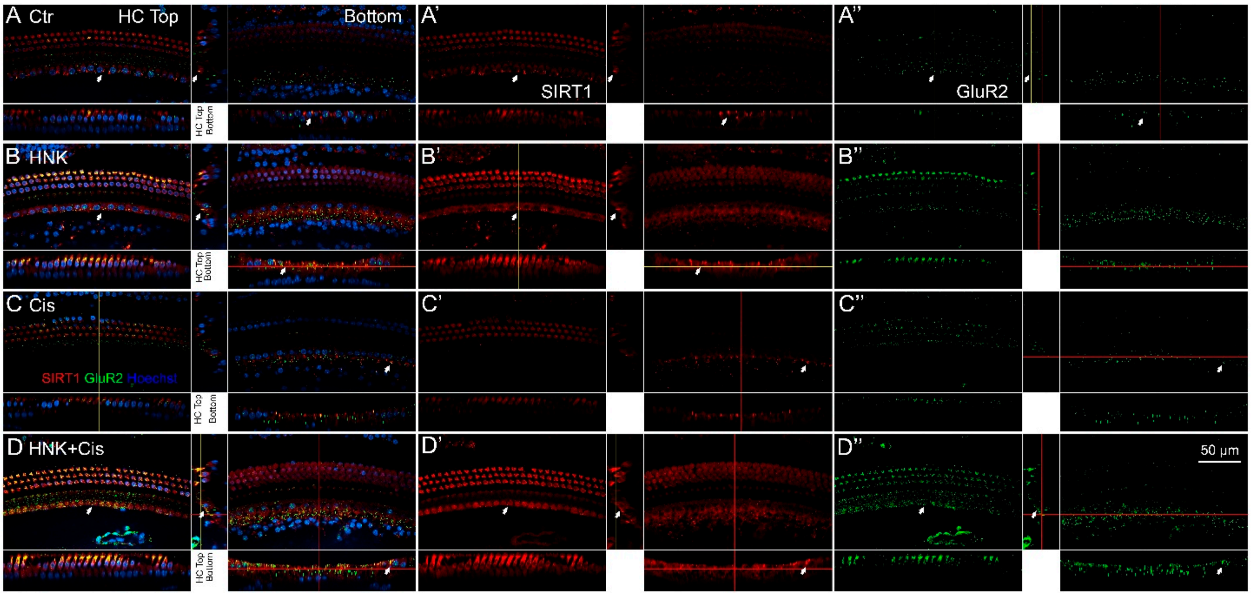

Figure S7. Cytoplasmic Sirt1 (red) and post-synaptic GluR2 (green) expression in the cochlea (frequency range: 19.1-36.5 kHz) after different treatments: (**A**) Control; (**B**) HNK10-Only; (**C**) Cis-Only; and (**D**) HNK10+Cis. Within each panel, surface plots and bottom orthogonal views focused on hair cell bodies (HC top, on the left) and nuclei/synapse (Bottom, on the right) were presented. Sirt1 (red) expression was observed in hair cells and was upregulated following honokiol treatment (**B**,**D**), while suppressed by cisplatin alone (**C**). Notably, distinct Sirt1 puncta were observed at the basal of the IHCs. White arrows indicate colocalization of these Sirt1 puncta with the postsynaptic marker GluR2 (green) at specific locations.

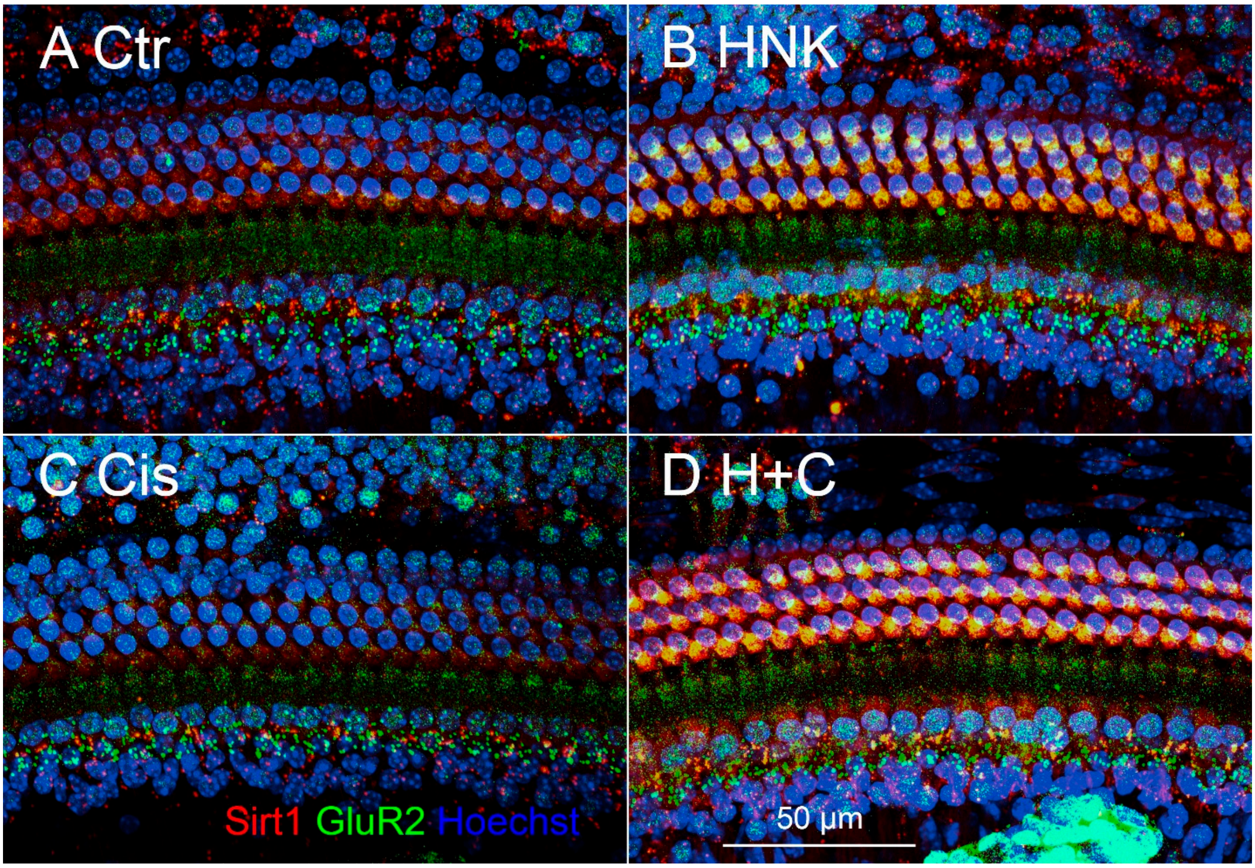

Figure S8. Projection of confocal image stacks for Sirt1 vs. GluR2 expression in the cochlea as shown in Figure S7 and Figure 8. (**A**) Control; (**B**) HNK10-Only; (**C**) Cis-Only; and (**D**) HNK10+Cis. Image stacks were processed in FIGI using the function of "3D Projection" in the "Stacks" sub-menu. The entire image stacks were projected onto a single image showing the brightest signal on each pixel, and therefore the images might be slightly distorted in terms of image size and spatial relationships among different structures and cells. Upregulated signals of Sirt1 in honokiol treated samples were shown most prominently in OHCs ((**B**) and (**D**)). Bright GluR2 puncta (green) indicated the location of the ribbon synapses. Overlapping Sirt1 and GluR2 puncta were observed in the area close to IHC nuclei.

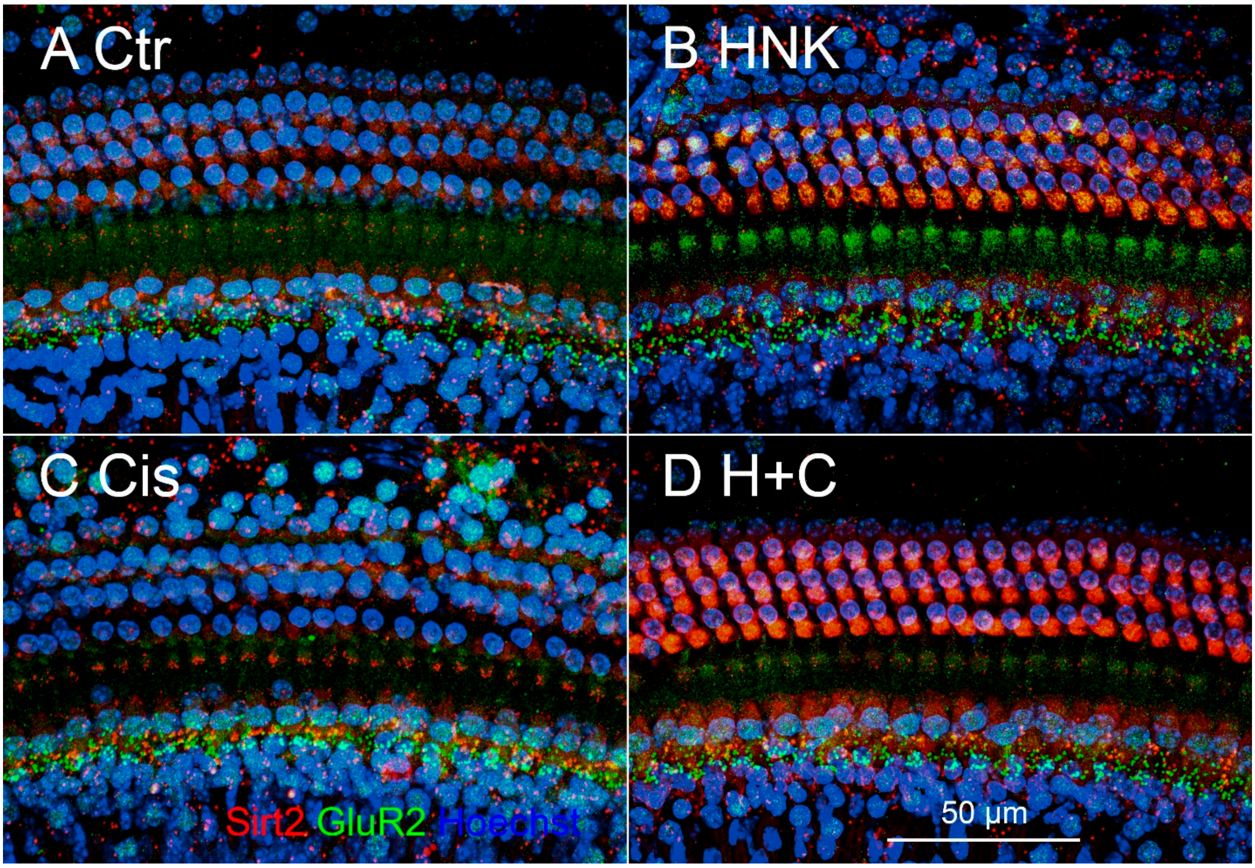

Figure S9. Cytoplasmic Sirt2 and post-synaptic GluR2 expression in the cochlea (frequency range: 9.5-19.1 kHz) after different treatments. (**A**) Control; (**B**) HNK10-Only; (**C**) Cis-Only; and (**D**) HNK10+Cis. Same 3D Projection of confocal image stacks as in Figure S8 was performed. Similar expression pattern as Sirt1 was observed for Sirt2, which was also upregulated by honokiol ((**B**) and (**D**)). Co-localization of Sirt2 and GluR2 puncta was also shown in vicinity to IHC nuclei.

Table S1. Number of animals survived after each cycle of treatment.

Treatment Group	Initial (Day 0)	Cycle 1 (Day 14)	Cycle 2 (Day 28)	Cycle 3 (Day 42)
Control	19	—	—	—
HNK10-Only	7	7	—	—
Cis-Only	15	15	12	7
HNK5+Cis	8	8	8	6
HNK10+Cis	10	10	10	8
HNK20+Cis	8	8	7	5
HNK20-Only *	8	8	8	8

* FVB wild type.
